# Tamoxifen mechanically deactivates hepatic stellate cells via the G protein-coupled estrogen receptor

**DOI:** 10.1038/s41388-018-0631-3

**Published:** 2018-12-21

**Authors:** Ernesto Cortes, Dariusz Lachowski, Alistair Rice, Stephen D. Thorpe, Benjamin Robinson, Gulcen Yeldag, David A. Lee, Leo Ghemtio, Krista Rombouts, Armando E. del Río Hernández

**Affiliations:** 1 Cellular and Molecular Biomechanics Laboratory, Department of Bioengineering, Imperial College London, London, SW7 2AZ United Kingdom; 2 Institute of Bioengineering, School of Engineering and Material Science, Queen Mary University of London, London, E1 4NS United Kingdom; 3 Drug Research Program, Division of Pharmaceutical Biosciences, Faculty of Pharmacy, University of Helsinki, FI-00014 Helsinki, Finland; 4 Regenerative Medicine and Fibrosis Group, Institute for Liver and Digestive Health, University College London, Royal Free Hospital, London, United Kingdom

**Keywords:** Myosin, Cancer microenvironment

## Abstract

Tamoxifen has been used for many years to target estrogen receptor signalling in breast cancer cells. Tamoxifen is also an agonist of the G protein-coupled estrogen receptor (GPER), a GPCR ubiquitously expressed in tissues that mediates the acute response to estrogens. Here we report that tamoxifen promotes mechanical quiescence in hepatic stellate cells (HSCs), stromal fibroblast-like cells whose activation triggers and perpetuates liver fibrosis in hepatocellular carcinomas. This mechanical deactivation is mediated by the GPER/RhoA/myosin axis and induces YAP deactivation. We report that tamoxifen decreases the levels of hypoxia-inducible factor-1 alpha (HIF-1α) and the synthesis of extracellular matrix proteins through a mechanical mechanism that involves actomyosin-dependent contractility and mechanosensing of tissue stiffness. Our results implicate GPER-mediated estrogen signalling in the mechanosensory-driven activation of HSCs and put forward estrogenic signalling as an option for mechanical reprogramming of myofibroblast-like cells in the tumour microenvironment. Tamoxifen, with half a century of safe clinical use, might lead this strategy of drug repositioning.

## Introduction

The G protein-coupled estrogen receptor (GPER) is a seven transmembrane G protein-coupled receptor (GPCR) that mediates the acute response to extracellular estrogens [1, 2]. Agonists for GPER include endogenous estrogens such as 17β-estradiol, as well as synthetic compounds, such as tamoxifen and fulvestrant. Tamoxifen is a GPER agonist that has been used in clinics for more than 50 years as hormonal therapy for breast cancer based on the classical genomic estrogen receptor (ER) signalling pathway, unrelated to GPER. Interestingly, tamoxifen has also been used in women at risk of developing breast cancer and has been observed to reduce mammographic density and fibrosis [3, 4]. Due to its established pharmacology and non-toxicity, tamoxifen is well positioned to lead our efforts in exploring novel modes of action for this drug and investigating the possible clinical benefits of GPER-mediated estrogen signalling.

Hepatocellular carcinoma (HCC) is the most common form of primary liver cancer and, regardless of aetiology, occurs predominantly in patients with cirrhosis, which is characterised by excessive extracellular matrix (ECM) deposition that presents the ideal environment for promoting tumour formation. In this environment, hepatocyte necrosis, inflammation, oxidative stress and hypoxia are responsible for genetic alterations and deregulation of signalling pathways that promote HCC development [5–7]. It is also known that inflammation in the stroma in HCC can be modulated by estrogens [8].

Hepatic stellate cells (HSCs) are stroma resident mesenchymal myofibroblast-like cells [9] which initiate and modulate liver fibrosis by regulating the chemical [10] and mechanical [11] composition of the ECM. HSCs, like other myofibroblast-like cells [12], are highly responsive to mechanical cues, requiring a stiff microenvironment to become activated and therefore initiate and perpetuate fibrosis. They achieve this by (i) activating their contractile apparatus to apply endogenous forces to the ECM, and (ii) mechanosensing the rigidity from their surroundings [13, 14]. Both processes, cell contractility and mechanosensing, rely on activation of the small GTPase RhoA [15, 16]. RhoA is essential in maintaining the activated phenotype of HSCs, by ensuring cell contractility through regulation of ROCK and other modulators of actomyosin [17].

Here we report that tamoxifen induces the mechanical deactivation of HSCs via a previously unidentified mechanism that involves the GPER/RhoA/myosin axis. This inhibits activation of YAP (Yes-associated protein) and durotaxis in HSCs. We also show that cell contractility and ECM rigidity regulate the levels of hypoxia-inducible factor 1 alpha (HIF-1α) and lysyl oxidase (LOX) in HSCs, and that tamoxifen suppresses force-mediated regulation of both HIF-1α and LOX. HIF-1α is fundamental for cell survival in hypoxic conditions [18], as the LOX family regulates collagen crosslinking and ECM architecture and is therefore required for hypoxia-induced metastasis.

## Results

### Tamoxifen treatment reduces myosin activation in HSCs via GPER/RhoA signalling

Actomyosin contractility is a key characteristic of activated HSCs, allowing mechanotransduction and force generation. This behaviour is adaptive in wound healing but promotes fibrosis in HCC [11]. Regulation of actomyosin contractility is achieved through phosphorylation of the regulatory protein myosin light chain 2 (MLC-2). Tamoxifen is a 17β-estradiol mimetic that activates GPER, and has been well characterised in its ability to selectively modulate estrogen receptors [19]. We used immunofluorescence staining to confirm the presence of GPER and the canonical estrogen receptors alpha and beta (ER-α and ER-β) in HSCs (Supplementary Fig S1). We also confirmed the levels of expression of GPER in HSCs using immunoblotting/immunofluorescence and GPER knockdown/overexpression (Supplementary Fig S2-S4). To assess actomyosin contractility, we determined the levels of active phosphorylated MLC-2 (pMLC-2), as well as the total MLC-2, in response to 10-day treatment with tamoxifen. We also included conditions with antagonists against ER and GPER to explore which receptor tamoxifen acted through. We used the selective ER antagonist (ICI182780) [20] and the specific GPER antagonist G15 [21]. For this experiment and the subsequent ones, ICI182780 and G15 were used simultaneously with tamoxifen treatment.

**Fig. 1:**
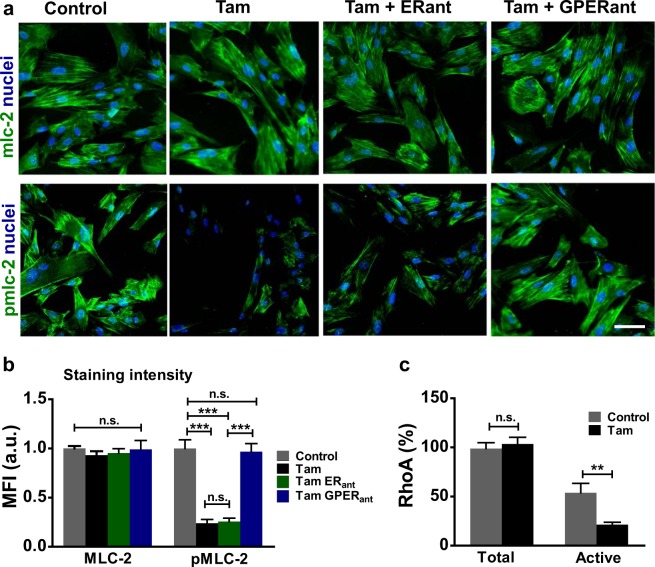
GPER activation in HSCs suppresses activation of MLC-2. **a** Representative images for immunofluorescence staining of HSCs, scale bar 50 µm. **b** Quantification of immunofluorescence staining for panel a. MFI mean fluorescence intensity, 12 fields of views with approximately 20 cells per field per condition. **c** Quantification of total and active RhoA, expressed as percentage of the total RhoA in the control condition, three biological samples analysed in three different experiments. All histogram bars represent mean ± sem, **P < 0.01,***P < 0.001. Anova and Tukey’s test for b and *t*-test for (**c)**. Three experimental replicates in all panels

Across all four conditions (control, tamoxifen, tamoxifen + ER antagonist, tamoxifen + GPER antagonist), the staining intensity for MLC-2 remained constant, indicating that protein expression was unchanged following treatment (Fig. 1a, b). Levels of pMLC-2 were significantly reduced in the tamoxifen and tamoxifen + ER antagonist conditions compared to control, indicating that tamoxifen greatly reduces MLC-2 phosphorylation, but does not act through the nuclear estrogen receptors. Conversely, tamoxifen + GPER antagonist showed pMLC-2 staining intensity comparable to the control condition, suggesting that tamoxifen achieves inhibition of MLC-2 activation through GPER (Fig. 1a, b).

To further confirm the specific role of GPER, we performed experiments with siRNA to knockdown GPER expression, in combination with tamoxifen or the estrogen 17β-estradiol (E2). Treatment was performed for 72 h since GPER knockdown with siRNA is only stable for this amount of time. We observed that 72-h treatment of HSCs with either tamoxifen or E2 led to a decrease in pMLC-2 without affecting MLC-2 abundance, similar to 10-day tamoxifen treatment. Knockdown of GPER with siRNA GPER showed abundance of pMLC-2 comparable to the control condition, even in the presence of tamoxifen or E2 (Supplementary Fig S5). This demonstrates that estrogenic signalling, instigated by either tamoxifen or E2, acts through GPER to reduce MLC-2 phosphorylation. Likewise, we assessed the effect of tamoxifen on the activation of MLC-2 after 24-h treatment and observed that while the total levels of MLC-2 were kept constant, pMLC-2 were significantly decreased in the tamoxifen group and this effect was mediated by GPER (Supplementary Fig S6).

RhoA lies upstream of MLC-2 and controls MLC-2 activation [22]. We quantified the levels of total RhoA and active RhoA levels in HSCs under tamoxifen treatment. Both control and 10 day tamoxifen treated conditions showed similar levels of total RhoA. Control cells exhibited active RhoA levels of around 50% of total RhoA, significantly higher than in the tamoxifen treated cells where active RhoA levels were around 20% (Fig. 1c). Taken together, these data suggest that tamoxifen reduces myosin activation via the GPER/RhoA/MLC-2 axis.

### GPER activation in HSCs impairs force generation and increases cell compliance

**Fig. 2:**
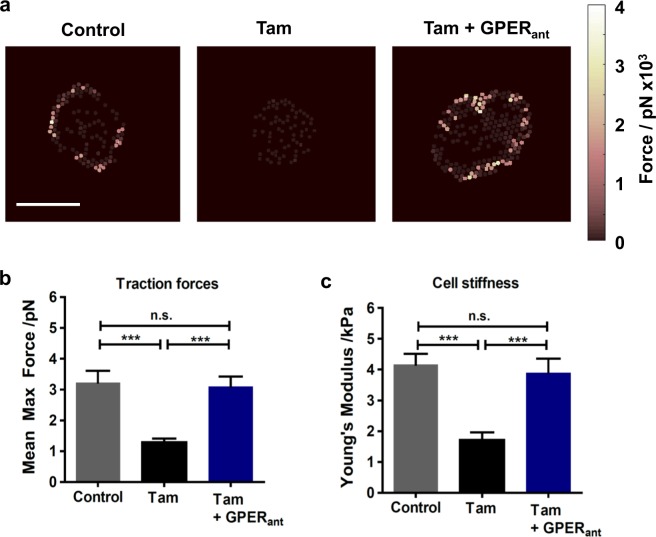
Tamoxifen treatment impairs traction forces and increases cell compliance in HSCs. **a** Heat maps representing forces applied by HSCs on top of pillar arrays, scale bar 20 µm. **b** Quantification of average forces applied by HSCs on pillars. *n* = 39 cells (control), 34 cells (tam) and 30 cells (tam and GPER antagonist). **c** Quantification of cell compliance with atomic force microscopy. Cantilevers used had a 15 µm polystyrene bead attached. *n* = 60 cells (control), 42 cells (tam) and 90 cells (tam and GPER antagonist). Mann–Whitney test for significance, ***P < 0.001. All histogram bars represent mean ± sem, **P < 0.01,***P < 0.001. Three experimental replicates in all panels

To further assess the effects of tamoxifen on cell contractility, we used elastic pillars as a form of traction force microscopy. This technique assesses the individual force applied to fibronectin-coated polydimethylsiloxane pillars during cell spreading. Using the deflection of each pillar in contact with the cell and the known Young’s modulus of each pillar in the array, quantitative analysis of force generation was achieved. We report the value of mean maximum force, calculated from the mean value of the maximum force experienced by each pillar with cellular contact during the time of analysis. Control HSCs generated a mean maximum force of around 3.2 nN, and following 10-day tamoxifen treatment, this mean maximum force was significantly reduced to around 1.2 nN. When a GPER antagonist was present alongside tamoxifen, the mean maximum force returned to a value comparable to control and significantly higher than tamoxifen alone (Fig. 2a, b). Both 72-h tamoxifen treatment and E2 treatment also significantly reduced traction forces in HSCs, but force generation was rescued in the presence of siRNA against GPER. Additionally, G1, a specifically designed GPER agonist [23], reduced traction forces, but did not with GPER knockdown (Supplementary Fig S7). These results indicate that GPER regulates cell traction forces, with tamoxifen, E2 and G1 all acting as GPER agonists.

The ability of cells to generate force is also dependent on their rheological properties such as cell stiffness [24, 25]. Drugs that disrupt the cytoskeleton are known to inhibit the activated phenotype of HSCs [26] and these types of drugs have also been shown to decrease cell stiffness [27]. We used atomic force microscopy (AFM) indentation of HSCs seeded on fibronectin-coated fluorodishes to determine cell elasticity. By indenting the cells at points between the nucleus and the cell edge, we ensured that our analysis would accurately assess the contribution of the cytoskeleton to cell elasticity, and would be unaffected by the nucleus or the underlying substrate.

We observed that control HSCs had a Young’s modulus around 4.1 kPa, and this was significantly reduced to around 1.7 kPa with 10-day tamoxifen treatment. However, with a GPER antagonist, tamoxifen was unable to reduce the Young’s modulus, and cells had an average Young’s modulus similar to the control condition (Fig. 2c). 72-h treatment with either tamoxifen, E2 or G1 reduced cell stiffness, but not in the presence of siRNA against GPER (Supplementary Fig S7).

### GPER activation reduces HSC mechanosensing and YAP activation

**Fig. 3:**
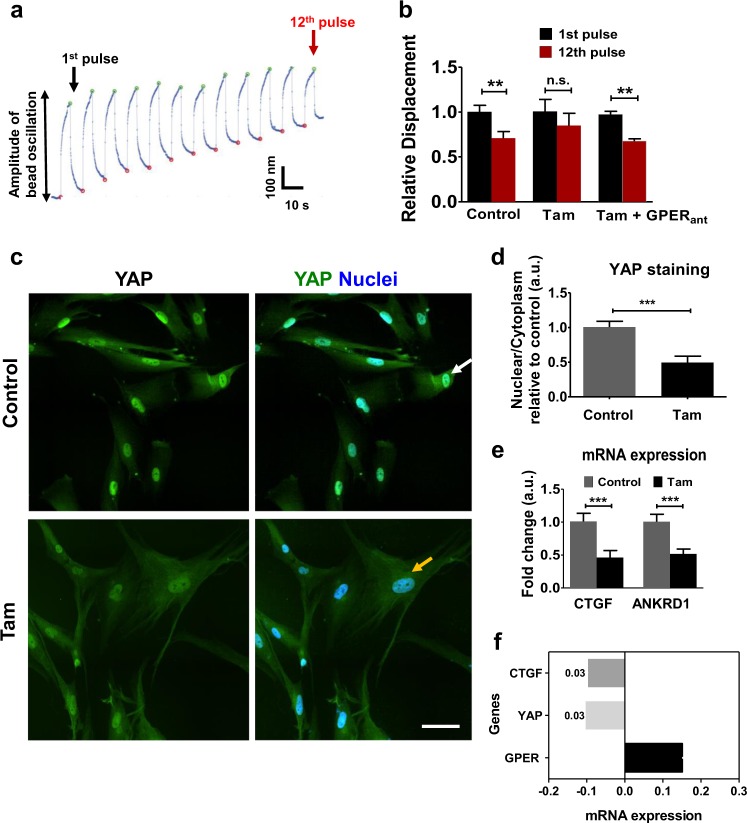
Tamoxifen treatment suppresses mechanosensing and YAP activation in HSCs. **a** Representative trace that shows the decrease in the amplitude of oscillation of a bead attached to a cell that can sense external mechanical stimuli (mechanosensing). **b** Histogram shows relative bead displacement for the first and last pulse, *n* = 25 cells (control), 20 cells (tam) and 21 cells (tam and GPER antagonist). **c** Representative images for immunofluorescence staining of HSCs, scale bar 20 µm. The white arrow indicates YAP nuclear localization, whereas the yellow arrow shows reduced nuclear YAP localization**. d** Quantification of YAP nuclear/cytoplasmic ratio, 16 control cells and 16 tamoxifen cells. **e** qPCR levels of YAP downstream genes CTGF and ANKRD1, normalized to RPLP0 and relative to control, three biological samples analysed in three different experiments. **f** Correlation GPER/YAP/CTGF expressions from TCGA database. Data from 492 patients. *t*-test, *P < 0.05, **P < 0.01,***P < 0.001. Three experimental replicates in all cases

We used magnetic tweezers microrheology to assess the ability of HSCs to respond to external mechanical forces, as would be experienced surrounded by a rigid stroma. Fibronectin-coated magnetic beads were attached to cells, and 12 consecutive pulses of equal force were applied with magnetic tweezers. Cells that displayed mechanosensitivity showed a reduction in bead displacement as the cytoskeleton reinforced following force application (Fig. 3a).

We observed that control HSCs showed mechanosensitivity, significantly reducing the displacement of the bead on the 12th pulse to 71% of the displacement measured on the 1st pulse. With 10 day tamoxifen treatment, the 12th pulse had 85% of the displacement measured on the 1st pulse, a value not significantly different from its first pulse displacement, indicating a reduction in mechanotransduction. Using the GPER antagonist G15, mechanotransduction was restored to control levels, with the 12th pulse at 68% compared to the 1st pulse (Fig. 3b).

The transcriptional regulator YAP is a key cellular mechanotransducer, converting external mechanical signals into changes in gene expression through its translocation to the nucleus [28]. YAP has further been shown to be essential in the mechanosensitive phenomenon of durotaxis in HSCs [29]. We stained control and 10-day tamoxifen-treated HSCs for YAP and assessed the intensity of staining in both the nucleus and cytoplasm. The ratio between these intensity values represents the level of YAP translocation to the nucleus and therefore activation. Control HSCs showed increased YAP nuclear localisation compared to 10-day tamoxifen-treated HSCs, suggesting that tamoxifen reduces levels of YAP mediated mechanotransduction (Fig. 3c, d). Seventy-two-hour treatment of HSCs with either tamoxifen or E2 also led to decreased YAP nuclear localisation, with GPER knockdown rescuing localisation to that of control HSCs (Supplementary Fig S8), indicating the specific role of GPER in estrogen-mediated YAP deactivation. The expression of the downstream YAP target genes CTGF and ANKRD1 were reduced in 10 day tamoxifen treated HSCs, in concurrence with the immunofluorescence data (Fig. 3e). We then performed correlation analysis for the expression profiles of the genes GPER, YAP and CTGF using the TCGA (Cancer Genome Atlas) database, and found that GPER expression in HCC patients negatively correlates with the expression of YAP and CTGF (Fig. 3f).

### Tamoxifen treatment induces HSC deactivation

Force generation and mechanotransduction are the two pillars required for maintenance of the activated phenotype of HSCs, similar to other myofibroblast-like cells [13, 30]. Since we observed tamoxifen to inhibit these mechanical properties, we assessed whether tamoxifen could promote HSC deactivation. We used immunofluorescence and qPCR to assess levels of α-SMA and vimentin, both markers of the activated phenotype. We observed a significant decrease in both α-SMA and vimentin with tamoxifen treatment at both the protein (Supplementary Fig S9) and mRNA (Supplementary Fig S10) levels.

With GPER knocked down with siRNA for 72 h, we observed no effect of tamoxifen in reducing the levels of both markers of quiescence in HSCs (α-SMA and vimentin), though 72 h treatment by tamoxifen did decrease these levels. Likewise, treating HSCs with 17 β-estradiol also downregulated the expression of α-SMA and vimentin to levels comparable to those observed in the tamoxifen group (Supplementary Fig S9). These results support the notion that tamoxifen promotes mechanical deactivation in HSCs through GPER/RhoA signalling.

### Tamoxifen treatment suppresses ECM protein production

**Fig. 4:**
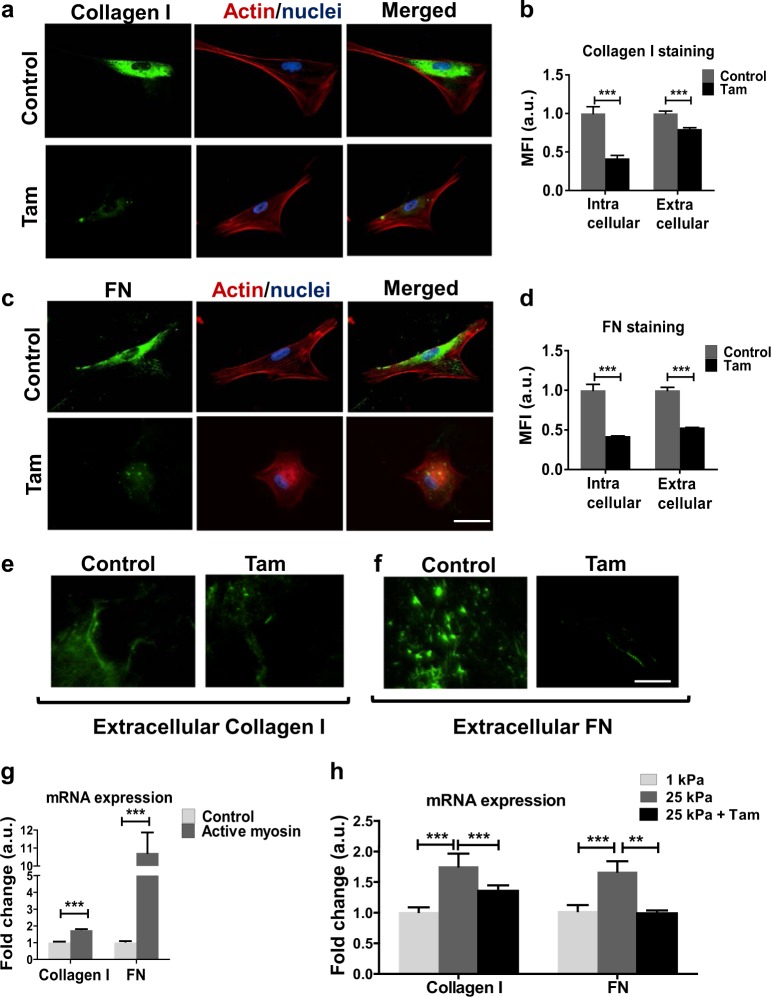
Tamoxifen treatment inhibits the synthesis and secretion of the ECM proteins collagen-I and fibronectin (FN). **a**, **c** Representative images for immunofluorescence staining of HSCs, scale bar 50 µm. **b, d** Quantification of immunofluorescence staining for (**a**,**c**,**e** and **f).** **b** 16 control cells and 14 tamoxifen cells. **d** 20 control cells and 14 tamoxifen cells. **e, f** Representative images for immunofluorescence images of secreted collagen-I and FN. **g** qPCR levels of collagen-I and FN in HSCs, normalized to RPLP0 (60 S acidic ribosomal protein P0) and relative to control. **h** qPCR levels of collagen-I and FN in HSCs, normalized to RPLP0 and relative to 1 kPa, *t*-test for b, d and g; and Anova and Tukey’s test for (**h**). **g**, **h** three biological samples analysed in three different experiments. All histogram bars represent mean ± sem, *P < 0.05, **P < 0.01,***P < 0.001. Three experimental replicates in all cases

A key role of activated HSCs in promoting further fibrosis and disease development is their ability to produce high levels of ECM proteins for secretion into their microenvironment. Collagen-I and fibronectin are abundant proteins within the ECM, playing critical roles in the organisation and structural integrity of the environment, and when overexpressed, can contribute to a pro-tumour microenvironment [31]. We used immunofluorescence to determine both the intracellular expression, and the extracellular secretion, of both collagen-I and fibronectin. We observed that 10-day tamoxifen treatment significantly reduced the intracellular and extracellular levels of both collagen-I (Fig. 4a, b, e) and fibronectin (Fig. 4c, d, f). 72-h tamoxifen treatment also reduced intracellular collagen-I and fibronectin levels, but could be rescued with GPER knockdown. E2 treatment for 72-h also showed the same trend (Supplementary Figure S11).

Due to the highly contractile phenotype of activated HSCs, and their role in ECM protein production, we assessed how enhancing contractile ability influenced collagen-I and fibronectin expression. We transfected control HSCs with constitutively active myosin-2 (pMLC-2) to increase cell contractility, and we observed significant increases in expression of both collagen-I and fibronectin (Fig. 4g), suggesting a mechanical basis to transcriptional regulation of both ECM proteins by HSCs.

We also assessed how changes in matrix rigidity, achieved through fabrication of different rigidity polyacrylamide (PAA) gels for cell culture, could change the production of collagen-I and fibronectin. A 1 kPa gel, which approximates the rigidity of healthy liver [32], was used as a soft substrate and we observed that increasing this rigidity to 25 kPa gave significant increases in collagen-I and fibronectin mRNAs. We further observed that 10-day tamoxifen treated HSCs on 25 kPa gels showed mRNA levels of collagen-I and fibronectin comparable to the 1 kPa condition, i.e. significantly lower than the 25 kPa condition (Fig. 4h). This indicates that tamoxifen inhibits the mechanical signalling pathway that connects external rigidity and increased ECM deposition. Collectively these results show that increased contractility and ECM stiffness trigger a transcriptional increase in both collagen-I and fibronectin in HSCs, and that tamoxifen inhibits this force-mediated activation.

### Tamoxifen treatment mechanically inhibits the HIF-1α/LOX and HIF-1α/LOX-L2 axes

Liver fibrosis in HCC, along with excess consumption of oxygen by hepatocytes, leads to tissue hypoxia, and the survival of cells becomes dependent on expression of HIF-1α [33]. Hypoxia, through HIF-1α, can regulate expression of ECM protein genes, such as fibronectin [34] and collagen-I [35]. HIF-1α has many other downstream targets, including members of the lysyl oxidase (LOX) family. Lysyl oxidases are copper-dependent enzymes that have fundamental roles in ECM organization in cancer. For instance, LOX is essential in hypoxia driven metastasis [36] and LOX-L2 is involved in ECM remodelling in fibrosis [37].

Mechanical induction of HIF-1α has also been observed in endothelial cells exposed to low shear stress [38], and in the myocardium in response to mechanical stress [39]. While hypoxia is the most common method of HIF-1α activation, upregulation of HIF-1α expression has also been seen in the presence of oxygen, with GPCRs on the cell surface responding to microenvironmental cues [40].

**Fig. 5:**
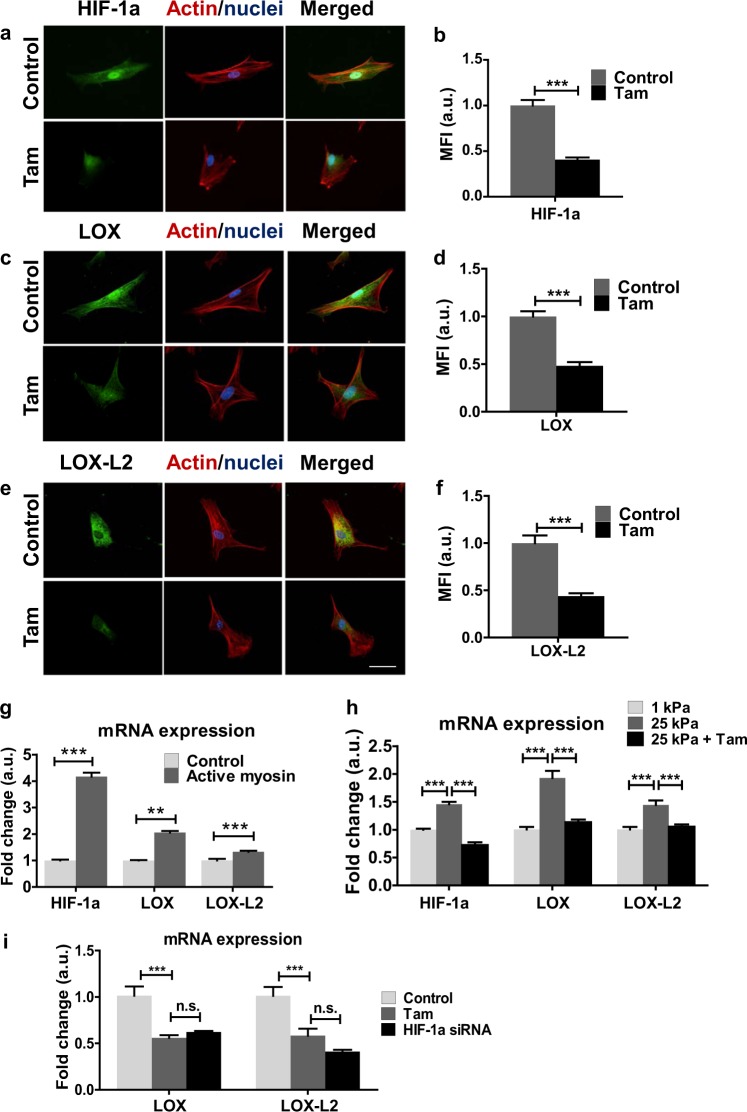
Tamoxifen treatment mechanically inhibits the HIF-1A/LOX and HIF-1A/LOX-L2 axes. **a**, **c** and **e** Representative images for immunofluorescence staining of HSCs. **b**, **d**, **f** Quantification of immunofluorescence staining for (**a**, **c** and **e**). Scale bar is 50 µm for all panels. **b** 20 control cells and 20 tamoxifen cells. **d** 12 control cells and 14 tamoxifen cells. **f** 12 control cells and 14 tamoxifen cells. **g** qPCR levels of HIF-1A, LOX and LOX-L2 in HSCs, normalized to RPLP0 (60 S acidic ribosomal protein P0) and relative to control. **h** qPCR levels of HIF-1A, LOX and LOX-L2 in HSCs, normalized to RPLP0 and relative to 1 kPa. **i** qPCR levels of LOX and LOX-L2 in HSCs, normalized to RPLP0 and relative to control. Three biological samples analysed in three different experiments. All histogram bars represent mean ± sem, *P < 0.05, **P < 0.01,***P < 0.001. Three experimental replicates in all cases. *t*-test for (**b**, **d** and **f)**, and Anova and Tukey’s test for (**g**, **h** and **i)**

We observed that levels of HIF-1α are reduced in HSCs following 10-day tamoxifen treatment (Fig. 5a, b). Furthermore, levels of LOX and LOX-L2 are also reduced with tamoxifen (Fig. 5c–f), suggesting that the ability of HSCs to cross-link collagen fibres in the ECM may be affected by tamoxifen treatment. Similarly, 72-h treatment by tamoxifen or E2 reduced levels of HIF-1α, LOX and LOX-L2, but no reduction was seen with GPER knockdown (Supplementary Figure S12).

Notably, we observed that the levels of HIF-1α, LOX and LOX-L2 are responsive to mechanical cues independent of tamoxifen-mediated signalling. The mRNA expression of these proteins is increased following transfection of HSCs with active myosin-2 (Fig. 5g). Similarly, the culturing of HSCs on polyacrylamide gels of differing rigidities also affected mRNA production. Compared to a 1 kPa substrate, HSCs cultured on a 25 kPa substrate showed a significantly increased expression of HIF-1α, LOX and LOX-L2 (Fig. 5h). This suggests that mechanotransduction alone can drive processes that promote survival under hypoxic conditions.

When tamoxifen was added to HSCs cultured on the stiff 25 kPa substrate for 10 days, levels of HIF-1α, LOX and LOX-L2 were reduced, becoming equivalent to the levels of these species on the soft 1 kPa substrate. To gain mechanistic insight into tamoxifen-induced downregulation of LOX, LOX-L2 and fibronectin, we used HIF-1α siRNA to knockdown HIF-1α expression. We observed that the mRNA levels of LOX, LOX-L2 and fibronectin, when treated with HIF-1α siRNA, were equivalent to the mRNA levels seen with tamoxifen treatment (Fig. 5i and Supplementary Fig S13). When taken together these results suggest that tamoxifen decreases LOX, LOX-L2 and fibronectin expression via HIF-1α, and that the effect of tamoxifen on HIF-1α levels is mechanically regulated by reducing myosin-2 dependent HSCs contractility and tissue stiffness.

### Tamoxifen impairs directed migration via GPER signalling

HSCs have been observed to migrate up a stiffness gradient, a process also known as durotaxis and this has been suggested to be a further step in the perpetuation of fibrosis in the liver [29]. Since this process was shown to be highly dependent on mechanotransduction, and our results here have shown tamoxifen to inhibit mechanotransduction through GPER, we investigated the ability of tamoxifen to inhibit HSC durotaxis.

**Fig. 6:**
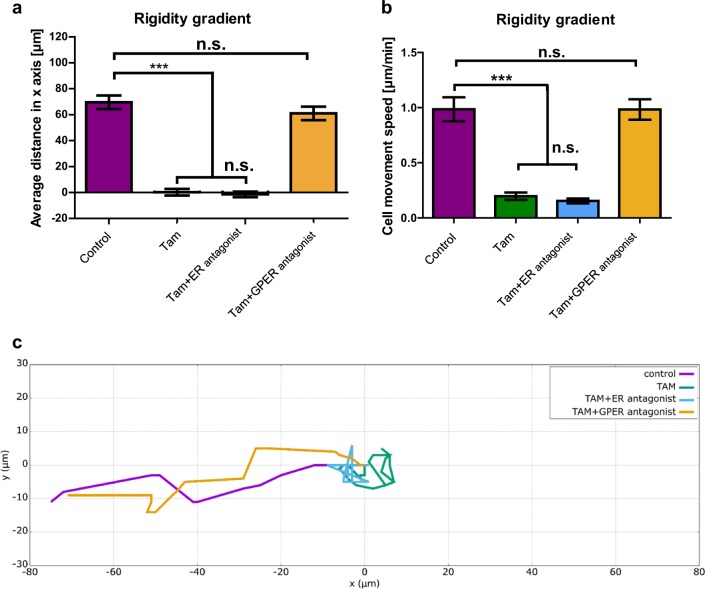
Tamoxifen treatment inhibits HSC durotaxis via GPER signalling. **a** Average cell movement distance on the soft-stiff rigidity gradient compared to single rigidity soft and stiff substrates presented as an average displacement (positive values indicate directed movement towards stiff substrate, negative values towards soft substrate and 0 indicates random movement. *n* = 3 independent experiments. **b** Cell movement speed on the soft-stiff rigidity gradient compared to single rigidity soft and stiff substrates. **c** Representation of the average displacements of HSCs. Quantification was done for 75 cells. Three experimental replicates in all cases. Results are expressed as mean ± sem. Anova and Tukey’s post hoc tests were used for the analysis

We prepared PAA gels of dual rigidity to assess HSC durotaxis in vitro following a protocol previously described [29]. On these gels, control HSCs moved an average distance in the X-axis (up the stiffness gradient) of around 70 µm over 5 ½ h, with an average speed of 0.99 µm/min along their individual paths. Ten- day tamoxifen treated cells and tamoxifen combined with an ER antagonist treated cells both showed no durotaxis, with significantly reduced cell movement speeds of 0.20 µm/min and 0.15 µm/min, respectively. When tamoxifen was combined with a GPER antagonist, durotaxis was rescued (average distance in X-axis = 61 µm over 5 ½ h) and cell speed became similar to the control condition (0.98 µm/min) (Fig. 6 and videos 1–4). Seventy-two-hour treatment of HSCs with tamoxifen or G1 also abrogated durotaxis behaviour, though GPER knockdown was able to rescue the tamoxifen treated cells, with durotaxis comparable to the control condition (Supplementary Figure S14 and videos 5–8). These combined results show that tamoxifen inhibits the ability of HSCs to migrate to stiffer substrates via GPER signalling.

## Discussion

Estrogens regulate a manifold of physiological and pathological processes and although endogenous estrogen is mainly derived from the ovaries in premenopausal women and mostly regarded as a female hormone [41], estrogen is also produced in other tissues, such as adipose tissues and arteries in both men and women [19, 42]. Until recently, the field of estrogens was dominated by studies that explored their transcriptional effects via nuclear estrogen receptors. However, the last decade has witnessed an explosion of interest in GPER-mediated estrogen signalling.

**Fig. 7:**
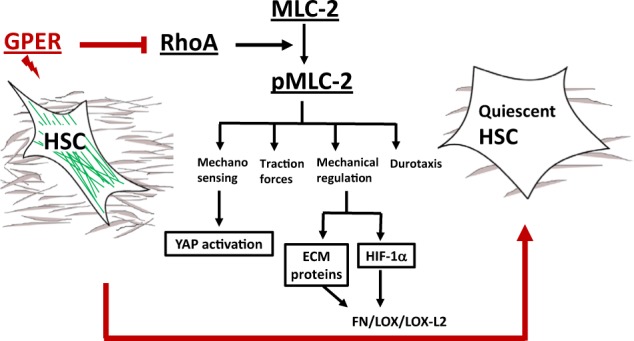
Model illustrating the mechanical effect of tamoxifen treatment in HSCs. Tamoxifen activates GPER and this downregulates the activity of RhoA, which in consequence decreases the levels of pMLC-2 (active form). The decrease in MLC-2 activation leads to suppressing mechanosensing, force generation and HSCs ability to mechanically regulate the synthesis of ECM proteins and HIF-1alpha

From our results, GPER comes to light as a comprehensive and effective mechanoregulator that targets the activation of fundamental proteins in cell mechanics, such as RhoA [22, 43] and MLC-2 to control force generation, mechanosensing and durotaxis in hepatic stellate cells. Increased levels of MLC-2 are required for the ability of stromal cells to remodel the ECM [44] to perpetuate fibrosis [45]. Likewise, high levels of active YAP, a mechanoresponsive transcriptional regulator [28], are indispensable for the activation of tumour-associated myofibroblasts in the stroma [46], and we show that YAP is downregulated in tamoxifen treated HSCs. Due to the similarity of activated HSCs to myofibroblasts, we posit that GPER is therefore likely to influence the mechanical properties of other stromal cells (Fig. 7).

The activation of HIF-1α is required for cell survival under hypoxic conditions. The dense stroma that surrounds solid tumours limits the accessibility of nutrients and oxygen to cells, promoting HIF-1α stabilisation [47]. The rapid growth of cancer cells within HCC also leads to high consumption of oxygen, further generating a hypoxic environment [48]. Our data suggest that HIF-1α is the unifying factor through which tamoxifen subsequently reduces the levels of LOX, LOX-L2 and fibronectin in HSCs. We suggest that HIF-1α may act as a converging point to mechanically regulate the adaptive response of HCC to hypoxia and the overall architecture of the tumour microenvironment. We propose that this mechanical regulation of HIF-1α by tamoxifen in an oxygen-independent manner may result in an effective reduction of cell fitness to cope with hypoxic condition in HSCs, and potentially in cancer cells as well, leading to decreasing fibrosis.

Development of fibrosis relies on positive feedback pathways, including mechanotransduction and ECM protein deposition [49] and durotaxis [29]. The directed migration of cells to stiffer fibrotic areas leads to further activation by mechanotransduction, leading to an increase in ECM protein production, which in turn, promotes a stiffer microenvironment [13] that might lead to increased chemoresistance in cancer cells [50]. Durotaxis can also play a role in facilitating cross-talk between cancer cells and activated stromal cells such as activated HSCs [51], and therefore the inhibition of directed migration by tamoxifen, combined with its ability to induce HSC quiescence, indicates the multiple ways in which tamoxifen could mechanically influence the tumor-stroma cross-talk.

Within the liver tissue, HSCs reside within the ECM, a mixture of scaffolding proteins secreted by HSCs, among other stromal cells. Interactions between cells are mostly through paracrine signalling, as well as interactions with the ECM proteins, rather than direct cell-cell interactions [9]. For our in vitro studies, we used culture-activated HSCs seeded on fibronectin-coated glass, a widely employed model, which recapitulates the activated phenotype in vivo with good approximation [10]. However, the behaviour we observe in vitro may well differ from that in vivo, where HSCs are influenced by factors secreted by cancer cells and other stromal cells, as well as the complex architecture of the ECM [9], which are not present in our in vitro setup. Further studies with HSCs are therefore necessary for a full comprehension of the role of GPER in vivo.

Our work lays the foundations for further studies that could directly influence therapeutic development. Tamoxifen is a widely used drug in clinics, with well-established pharmacodynamics [52] and safety [53], and due to the pleiotropic effects of estrogens and the commonality of GPCR signaling pathways, it is possible that tamoxifen regulates many genes involved in the function of myofibroblast-like cells such as activated HSCs or cancer associated fibroblasts. This could lead to development of stromal reprogramming strategies in which GPER agonists could modulate the fibrovascular stroma of HCC to increase vascular density and perfusion by reducing overall solid stress, achieved through inhibiting expression of collagen and fibronectin. This would increase intratumoral drug perfusion, while concurrently impeding the adaptive fitness of tumour and stromal cells to survive under hypoxic conditions (via HIF-1α) and thus promote widespread hypoxic necrosis.

## Materials and methods

### Cell culture and antibodies

Primary, culture-activated human hepatic stellate cells (HSCs), passage 3–6, (HHStec 5300; ScienCell, Carlsbad, CA, USA) were cultured in phenol red medium (DMEM-F12 HAM, cat. D6434, Sigma–Aldrich) supplemented with 10% foetal bovine serum (cat. 10500–064, Gibco), 1% penicillin/streptomycin (P4333, Sigma–Aldrich, USA) and 1% Fungizone R amphotericin (15290–026, Gibco). These cells were tested for mycoplasma contamination. Tamoxifen (Z-4-hydroxytamoxifen, cat. H7904 Sigma–Aldrich, USA) and 17 β-Estradiol (E2) (catalog number E8875, Sigma–Aldrich, USA) were prepared in ethanol (stock solution 100 μM). The specific ER antagonist (ICI182780) [20], GPER antagonist (G15) [21] and specifically designed GPER agonist [23] were purchased from Tocris (cat. 1047, 3678 and 3577, respectively). ICI182780, G1 and G15 were prepared in DMSO (stock solution 50 mM). To prevent any estrogenic effects from phenol red, during the treatment with tamoxifen, E2 or G1, HSCs were transferred to clear medium with no phenol red (DMEM-F12 HAM, cat. D8437, Sigma–Aldrich, USA) supplemented with 10% double charcoal stripped foetal calf serum—DCSS (cat. 02-46-850, First Link, Wolverhampton, UK), 1% penicillin/streptomycin (P4333, Sigma Aldrich, USA) and 1% Fungizone R Amphotericin (15290-026, Gibco, USA). For subsequent experiments media (without phenol red) and DCSS were used. In all experiments tamoxifen was used at 5 µM and E2 at 0.1 µM. GPER agonist G1 was used at 1 µM. ER and GPER antagonists (ICI182780 and G15) were added simultaneously with tamoxifen in all experiments; the concentration used for both was 1 µM. This range of concentrations have been used effectively in previous studies [54]. Tamoxifen treatment was done for 72 h or 10 days. E2 and G1 treatments were conducted for 72 h. Media was replenished every 72 h in all cases. For GPER and HIF-1α knockdowns, siRNA for GPER (Santa Cruz Biotechnology, cat. sc-60743) and siRNA for HIF-1A (cat. Sc-35561, from Santa Cruz Biotechnology USA), respectively, were used to transfect HSCs before the specific treatment. Human plasma fibronectin (FC010) was from Millipore USA. The followings are antibodies: MLC-2 (Millipore USA, MABT180, 1/200), pMLC-2 /Thr18/Ser19 (Cell Signaling USA, 3674, 1/200), total RhoA (Millipore USA, 04–822 USA, WB 1/1000), pRhoA (Abcam UK, ab41435, WB 1/100), YAP (Santa Cruz Biotechnology USA, sc-101199, 1/100), collagen-I (Abcam UK, ab34710, 1/100), fibronectin (Abcam UK ab2413, 1/500), HIF-1 alpha (Abcam UK, ab2185 1/200), LOX (Santa Cruz Biotechnology USA, sc-373950, 1/100), LOX-L2 (Santa Cruz Biotechnology USA, sc-66950, 1/50), αSMA (Abcam UK, ab7817, 1/200), Vimentin (DAKO UK, M0725, 1/100), GPER (Abcam UK, ab39742, 1/1000 and 1/2500), GPER (Abcam UK, ab154069, 1/1000), anti-Mouse HRP (Invitrogen USA, 626580, 1/2,000), anti-Rabbit HRP (Abcam UK, ab137914, 1/200) and a-Mouse 488 (Invitrogen USA, A11029, 1/400). The GPER plasmid used to overexpress GPER was obtained from Sino Biological, UK (catalog number HG11264-ACG), and a stop codon was inserted between the GPER and GFP sequences by site directed mutagenesis. pEGFP-MRLC1 (constitutively active MLC-2) was a gift from Tom Egelhoff, Addgene USA plasmid #35680.

### Immunofluorescence staining

Cell immunofluorescence staining was done on coverslips coated with 10 μg ml^−1^ fibronectin (Gibco, phe0023). Following pertinent treatment cells were fixed with 4% paraformaldehyde (Sigma, P6148) in D-PBS (Sigma, D8537) for 10 min, and then blocked and permeabilized with 0.2% BSA–0.1%Triton (Sigma, T8787) in PBS for 30 min. After blocking, cells were incubated with primary antibodies prepared in blocking solution for 1 h at room temperature in a humidified chamber. Then, cells were washed in D-PBS and incubated with Alexa Fluor 488-conjugated secondary antibodies and Phalloidin (Invitrogen, A22283, 1/1,000 dilution) prepared in PBS for 30 min at room temperature. Finally, coverslips were washed in PBS and mounted in mounting reagent with 4,6-diamidino-2-phenylindole (Invitrogen, P36931). Immunofluorescent images were taken with Nikon Ti-e inverted microscope (Nikon, Kingston-upon-Thames, United Kingdom) with NIS elements software.

### RT–PCR

Total RNA was extracted using the RNeasy Mini kit (Qiagen, 74104) and 1 μg of total RNA was reverse-transcribed using the High-Capacity RNA-to-cDNA kit (Applied Biosystems, 4387406) according to the manufacturer’s instructions. qPCR was performed using the SYBR Green PCR Master Mix (Applied Biosystems, 4309155) with 100 ng cDNA input in 20 μl reaction volume. RPLP0 expression level was used for normalization as a housekeeping gene. The primer sequences were as follow: RPLP0: forward 5′-CGGTTTCTGATTGGCTAC-3′, and reverse 5′-ACGATGTCACTTCCACG-3′; CTGF: forward 5′-TTAAGAAGGGCAAAAAGTGC-3′, and reverse 5′-CATACTCCACAGAATTTAGCTC-3′; ANKDR1: forward 5′-TGAGTATAAACGGACAGCTC-3′, and reverse 5′-TATCACGGAATTCGATCTGG-3′; a-SMA: forward 5′CATCATGAAGTGTGACATCG-3′, and reverse 5′GATCTTGATCTTCATGGTGC-3′; Collagen-I: forward 5′-GCTATGATGAGAAATCAACCG-3′, and reverse 5′-TCATCTCCATTCTTTCCAGG-3′; fibronectin: forward 5′-CCATAGCTGAGAAGTGTTTTG-3′, and reverse 5′-CAAGTACAATCTACCATCATCC-3′; HIF-1A: forward 5′-AAAATCTCATCCAAGAAGCC-3′, and reverse 5′-AATGTTCCAATTCCTACTGC-3′; LOX: forward 5′-CAACATTACCACAGTATGGATG-3′, and reverse 5′-TAGTCACAGGATGTGTCTTC-3′; LOX-L2; forward 5′-GATGTACAACTGCCACATAG-3′, and reverse 5′-GACAGCTGGTTGTTTAAGAG-3′. All primers were used at 300 nM final concentration. The relative gene expression was analysed by comparative 2−ΔΔct method.

The procedures for the analysis of gene expression using TCGA data, traction forces using elastic pillars, cell mechanosensing, durotaxis, atomic force microscopy, GLISA and the statistical analysis can be found in supplementary methods.

## Supplementary information

Supplementary information guide

Supplementary Figures

Supplementary methods information

Supplementary file Video 1 Durotaxis control 10d

Supplementary file Video 2 Durotaxis tamoxifen 10d

Supplementary file Video 3 Durotaxis tamoxifen 10d + ER antagonist

Supplementary fileVideo 4 Durotaxis tamoxifen 10d + GPER antagonist

Supplementary file Video 5 Durotaxis control 72h

Supplementary fileVideo 6 Durotaxis tamoxifen 72h

Supplementary fileVideo 7 Durotaxis G1 72h

Supplementary file Video 8 Durotaxis tamoxifen 72h + siRNA GPER

Supplementary file Summary video

## Data Availability

All relevant data are available from the authors.

## References

[1] Zimmerman MA, Budish RA, Kashyap S, Lindsey SH (2016). GPER-novel membrane oestrogen receptor. Clin Sci.

[2] Revankar CM, Cimino DF, Sklar LA, Arterburn JB, Prossnitz ER (2005). A transmembrane intracellular estrogen receptor mediates rapid cell signaling. Science.

[3] Cuzick J, Warwick J, Pinney E, Warren RM, Duffy SW (2004). Tamoxifen and breast density in women at increased risk of breast cancer. J Natl Cancer Inst.

[4] Cuzick J, Sestak I, Cawthorn S, Hamed H, Holli K, Howell A (2015). Tamoxifen for prevention of breast cancer: extended long-term follow-up of the IBIS-I breast cancer prevention trial. Lancet Oncol.

[5] Balogh J, Victor D, Asham EH, Burroughs SG, Boktour M, Saharia A (2016). Hepatocellular carcinoma: a review. J Hepatocell Carcinoma.

[6] Fattovich G, Stroffolini T, Zagni I, Donato F (2004). Hepatocellular carcinoma in cirrhosis: incidence and risk factors. Gastroenterology.

[7] Sakurai T, Kudo M (2013). Molecular link between liver fibrosis and hepatocellular carcinoma. Liver Cancer.

[8] Shi L, Feng Y, Lin HF, Ma RN, Cai X (2014). Role of estrogen in hepatocellular carcinoma: is inflammation the key?. J Transl Med.

[9] Moreira RK (2007). Hepatic stellate cells and liver fibrosis. Arch Pathol Lab Med.

[10] Yin C, Evason KJ, Asahina K, Stainier DY (2013). Hepatic stellate cells in liver development, regeneration, and cancer. J Clin Invest.

[11] Carloni V, Luong TV, Rombouts K (2014). Hepatic stellate cells and extracellular matrix in hepatocellular carcinoma: more complicated than ever. Liver Int.

[12] Lachowski D, Cortes E, Pink D, Chronopoulos A, Karim SA, PM J (2017). Substrate rigidity controls activation and durotaxis in pancreatic stellate cells. Sci Rep.

[13] Wells RG (2005). The role of matrix stiffness in hepatic stellate cell activation and liver fibrosis. J Clin Gastroenterol.

[14] Cortes Ernesto, Lachowski Dariusz, Rice Alistair, Chronopoulos Antonios, Robinson Benjamin, Thorpe Stephen, Lee David A, Possamai Lucia A, Wang Haiyun, Pinato David J, del Río Hernández Armando E. (2018). Retinoic Acid Receptor-β Is Downregulated in Hepatocellular Carcinoma and Cirrhosis and Its Expression Inhibits Myosin-Driven Activation and Durotaxis in Hepatic Stellate Cells. Hepatology.

[15] Rodriguez-Hernandez Irene, Cantelli Gaia, Bruce Fanshawe, Sanz-Moreno Victoria (2016). Rho, ROCK and actomyosin contractility in metastasis as drug targets. F1000Research.

[16] Guilluy C, Swaminathan V, Garcia-Mata R, O’Brien ET, Superfine R, Burridge K (2011). The Rho GEFs LARG and GEF-H1 regulate the mechanical response to force on integrins. Nat Cell Biol.

[17] Soon RK, Yee HF (2008). Stellate cell contraction: role, regulation, and potential therapeutic target. Clin Liver Dis.

[18] Harris AL (2002). Hypoxia–a key regulatory factor in tumour growth. Nat Rev Cancer.

[19] Prossnitz ER, Barton M (2011). The G-protein-coupled estrogen receptor GPER in health and disease. Nat Rev Endocrinol.

[20] Howell A, Osborne CK, Morris C, Wakeling AE (2000). ICI 182,780 (Faslodex): development of a novel, “pure” antiestrogen. Cancer.

[21] Dennis MK, Burai R, Ramesh C, Petrie WK, Alcon SN, Nayak TK (2009). In vivo effects of a GPR30 antagonist. Nat Chem Biol.

[22] Somlyo AP, Somlyo AV (2000). Signal transduction by G-proteins, rho-kinase and protein phosphatase to smooth muscle and non-muscle myosin II. J Physiol.

[23] Bologa CG, Revankar CM, Young SM, Edwards BS, Arterburn JB, Kiselyov AS (2006). Virtual and biomolecular screening converge on a selective agonist for GPR30. Nat Chem Biol.

[24] Stamenovic D (2005). Effects of cytoskeletal prestress on cell rheological behavior. Acta Biomater.

[25] Wang N, Tolic-Norrelykke IM, Chen J, Mijailovich SM, Butler JP, Fredberg JJ (2002). Cell prestress. I. Stiffness and prestress are closely associated in adherent contractile cells. Am J Physiol Cell Physiol.

[26] Cui X, Zhang X, Yin Q, Meng A, Su S, Jing X (2014). Factin cytoskeleton reorganization is associated with hepatic stellate cell activation. Mol Med Rep.

[27] Kuznetsova TG, Starodubtseva MN, Yegorenkov NI, Chizhik SA, Zhdanov RI (2007). Atomic force microscopy probing of cell elasticity. Micron.

[28] Dupont S, Morsut L, Aragona M, Enzo E, Giulitti S, Cordenonsi M (2011). Role of YAP/TAZ in mechanotransduction. Nature.

[29] Lachowski Dariusz, Cortes Ernesto, Robinson Benjamin, Rice Alistair, Rombouts Krista, Del Río Hernández Armando E. (2018). FAK controls the mechanical activation of YAP, a transcriptional regulator required for durotaxis. The FASEB Journal.

[30] Chronopoulos A, Robinson B, Sarper M, Cortes E, Auernheimer V, Lachowski D (2016). ATRA mechanically reprograms pancreatic stellate cells to suppress matrix remodelling and inhibit cancer cell invasion. Nat Commun.

[31] Lu P, Weaver VM, Werb Z (2012). The extracellular matrix: a dynamic niche in cancer progression. J Cell Biol.

[32] Mueller S, Sandrin L (2010). Liver stiffness: a novel parameter for the diagnosis of liver disease. Hepatic Med: Evid Res.

[33] Ju C, Colgan SP, Eltzschig HK (2016). Hypoxia-inducible factors as molecular targets for liver diseases. J Mol Med.

[34] Krishnamachary B, Berg-Dixon S, Kelly B, Agani F, Feldser D, Ferreira G (2003). Regulation of colon carcinoma cell invasion by hypoxia-inducible factor 1. Cancer Res.

[35] Copple BL, Bai S, Burgoon LD, Moon JO (2011). Hypoxia-inducible factor-1alpha regulates the expression of genes in hypoxic hepatic stellate cells important for collagen deposition and angiogenesis. Liver Int.

[36] Erler JT, Bennewith KL, Nicolau M, Dornhofer N, Kong C, Le QT (2006). Lysyl oxidase is essential for hypoxia-induced metastasis. Nature.

[37] Cano A, Santamaria PG, Moreno-Bueno G (2012). LOXL2 in epithelial cell plasticity and tumor progression. Future Oncol.

[38] Feng S, Bowden N, Fragiadaki M, Souilhol C, Hsiao S, Mahmoud M (2017). Mechanical activation of hypoxia-inducible factor 1alpha drives endothelial dysfunction at atheroprone sites. Arterioscler Thromb Vasc Biol.

[39] Kim CH, Cho YS, Chun YS, Park JW, Kim MS (2002). Early expression of myocardial HIF-1alpha in response to mechanical stresses: regulation by stretch-activated channels and the phosphatidylinositol 3-kinase signaling pathway. Circ Res.

[40] Wilson GK, Tennant DA, McKeating JA (2014). Hypoxia inducible factors in liver disease and hepatocellular carcinoma: current understanding and future directions. J Hepatol.

[41] Deroo BJ, Korach KS (2006). Estrogen receptors and human disease. J Clin Invest.

[42] Nathan L, Shi W, Dinh H, Mukherjee TK, Wang X, Lusis AJ (2001). Testosterone inhibits early atherogenesis by conversion to estradiol: critical role of aromatase. Proc Natl Acad Sci USA.

[43] Haining AWM, Rahikainen R, Cortes E, Lachowski D, Rice A, von Essen M (2018). Mechanotransduction in talin through the interaction of the R8 domain with DLC1. PLoS Biol.

[44] Robinson BK, Cortes E, Rice AJ, Sarper M, Del Rio, Hernandez A (2016). Quantitative analysis of 3D extracellular matrix remodelling by pancreatic stellate cells. Biol Open.

[45] Sarper M, Cortes E, Lieberthal TJ, Del Rio Hernandez A (2016). ATRA modulates mechanical activation of TGF-beta by pancreatic stellate cells. Sci Rep.

[46] Calvo F, Ege N, Grande-Garcia A, Hooper S, Jenkins RP, Chaudhry SI (2013). Mechanotransduction and YAP-dependent matrix remodelling is required for the generation and maintenance of cancer-associated fibroblasts. Nat Cell Biol.

[47] Chen S, Sang N (2016). Hypoxia-inducible factor-1: a critical player in the survival strategy of stressed cells. J Cell Biochem.

[48] Chen C, Lou T (2017). Hypoxia inducible factors in hepatocellular carcinoma. Oncotarget.

[49] Humphrey JD, Dufresne ER, Schwartz MA (2014). Mechanotransduction and extracellular matrix homeostasis. Nat Rev Mol Cell Biol.

[50] Rice AJ, Cortes E, Lachowski D, Cheung BCH, Karim SA, Morton JP (2017). Matrix stiffness induces epithelial-mesenchymal transition and promotes chemoresistance in pancreatic cancer cells. Oncogenesis.

[51] Coulouarn C, Corlu A, Glaise D, Guenon I, Thorgeirsson SS, Clement B (2012). Hepatocyte-stellate cell cross-talk in the liver engenders a permissive inflammatory microenvironment that drives progression in hepatocellular carcinoma. Cancer Res.

[52] Mandlekar S, Hebbar V, Christov K, Kong A (2000). Pharmacodynamics of tamoxifen and its 4-hydroxy and n-desmethyl metabolites: activation of caspases and induction of apoptosis in rat mammary tumors and in human breast cancer cell lines. Cancer Res.

[53] Kellof G, Crowell J, Boone C, Steele V, Lubet R, Greenwald P (1994). Clinical development plan: tamoxifen. J Cell Biochem Suppl.

[54] Chakrabarti S, Davidge ST (2012). G-protein coupled receptor 30 (GPR30): a novel regulator of endothelial inflammation. PLoS ONE.

