# Supplementary material for: Tamoxifen mechanically deactivates hepatic stellate cells via the G protein-coupled estrogen receptor
Source: Oncogene. 2018 Dec 21;38(16):2910–22. doi: 10.1038/s41388-018-0631-3 (PMC6755965; doi:10.1038/s41388-018-0631-3)
Supplement: Supplementary file 1 — Supplementary information guide [file 41388_2018_631_MOESM1_ESM.docx]

**Tamoxifen mechanically deactivates hepatic stellate cells via the G protein-coupled estrogen receptor**

Ernesto Cortes^1*^, Dariusz Lachowski^1*^, Alistair Rice^1*^, Benjamin Robinson^1^, Léo Ghemtio^2^, Krista Rombouts^3^, and Armando E. del Río Hernández^1^

**^1^**Cellular and Molecular Biomechanics Laboratory, Department of Bioengineering, Imperial College London, London SW7 2AZ, United Kingdom

^2^Drug Research Program, Division of Pharmaceutical Biosciences, Faculty of Pharmacy, FI-00014 University of Helsinki, Finland

^3^Regenerative Medicine and Fibrosis Group, Institute for Liver and Digestive Health, University College London, Royal Free Hospital, London, United Kingdom

**Supplementary information guide**

- **Supplementary figures S1 to S10**

Supplementary Fig S1: Expression of G coupled protein estrogen receptor (GPER), estrogen receptor alpha (ER-α), and estrogen receptor beta (ER-β) in hepatic stellate cells

Supplementary Fig S2: Immunoblotting experiments to validate GPER expression in HSCs and human embryonic kidney (HEK) cells (negative control). GPER antibody ab35742.

Supplementary Fig S3: Immunoblotting experiments to validate GPER expression in HSCs and human embryonic kidney (HEK) cells (negative control). GPER antibody ab154069.

Supplementary Figure S4: Knockdown efficiency of siRNA GPER in HSCs (Immunofluorescence).

Supplementary Fig S5: MLC-2/pML-2 data for control, tamoxifen, E2, control + siRNA GPER, tamoxifen + siRNA GPER, E2 + siRNA GPER. In all cases, the treatment was done for 72h.

Supplementary Figure S6. 24h Tamoxifen treatment downregulates MLC-2 activation via GPER in hepatic stellate cells (HSCs).

Supplementary Fig S7: Traction forces and cell stiffness data for control, tamoxifen, G1, E2, control + siRNA GPER, tamoxifen + siRNA GPER, G1 + siRNA GPER, E2 + siRNA GPER. In all cases, the treatment was done for 72h.

Supplementary Fig S8: YAP nuclear localization data for control, tamoxifen, E2, control + siRNA GPER, tamoxifen + siRNA GPER, E2 + siRNA GPER. In all cases, the treatment was done for 72h.

Supplementary Fig S9: α-SMA and vimentin protein expression for control, tamoxifen, E2, control + siRNA GPER, tamoxifen + siRNA GPER, E2 + siRNA GPER. In all cases, the treatment was done for 72h.

Supplementary Fig S10: α-SMA and vimentin mRNA expression for control and tamoxifen (10 days).

Supplementary Fig S11: Collagen I and fibronectin levels of expression data for control, tamoxifen, E2, control + siRNA GPER, tamoxifen + siRNA GPER, E2 + siRNA GPER. In all cases, the treatment was done for 72h.

Supplementary Fig S12: HIF-1α, LOX, and LOX-L2 data for control, tamoxifen, E2, control + siRNA GPER, tamoxifen + siRNA GPER, E2 + siRNA GPER. In all cases, the treatment was done for 72h.

Supplementary Figure S13: Tamoxifen effect on fibronectin (FN) is mediated by HIF-1a.

Supplementary Fig S14: Durotaxis data for control, tamoxifen, G1, tamoxifen + siRNA GPER. In all cases, the treatment was done for 72h.

- **Supplementary methods information**

Methods information for the analysis of gene expression using TCGA data, traction forces using elastic pillars, cell mechanosensing, durotaxis, atomic force microscopy, GLISA, and the statistical analysis.

- **Supplementary videos information**

These videos correspond to the durotaxis experiments reported in figure 6 and Supplementary figure S10:

- Video 1 Durotaxis control 10 days
- Video 2 Durotaxis Tamoxifen 10 days
- Video 3 Durotaxis Tamoxifen 10 days + ER antagonist
- Video 4 Durotaxis Tamoxifen 10 days + GPER antagonist
- Video 5 Durotaxis Control 72h
- Video 6 Durotaxis Tamoxifen 72h
- Video 7 Durotaxis G1 72h
- Video 8 Durotaxis Tamoxifen 72h + siRNA GPER
