# Supplementary material for: Tamoxifen mechanically deactivates hepatic stellate cells via the G protein-coupled estrogen receptor
Source: Oncogene. 2018 Dec 21;38(16):2910–22. doi: 10.1038/s41388-018-0631-3 (PMC6755965; doi:10.1038/s41388-018-0631-3)
Supplement: Supplementary file 2 — Supplementary Figures [file 41388_2018_631_MOESM2_ESM.pdf]

Cortes *et al*.

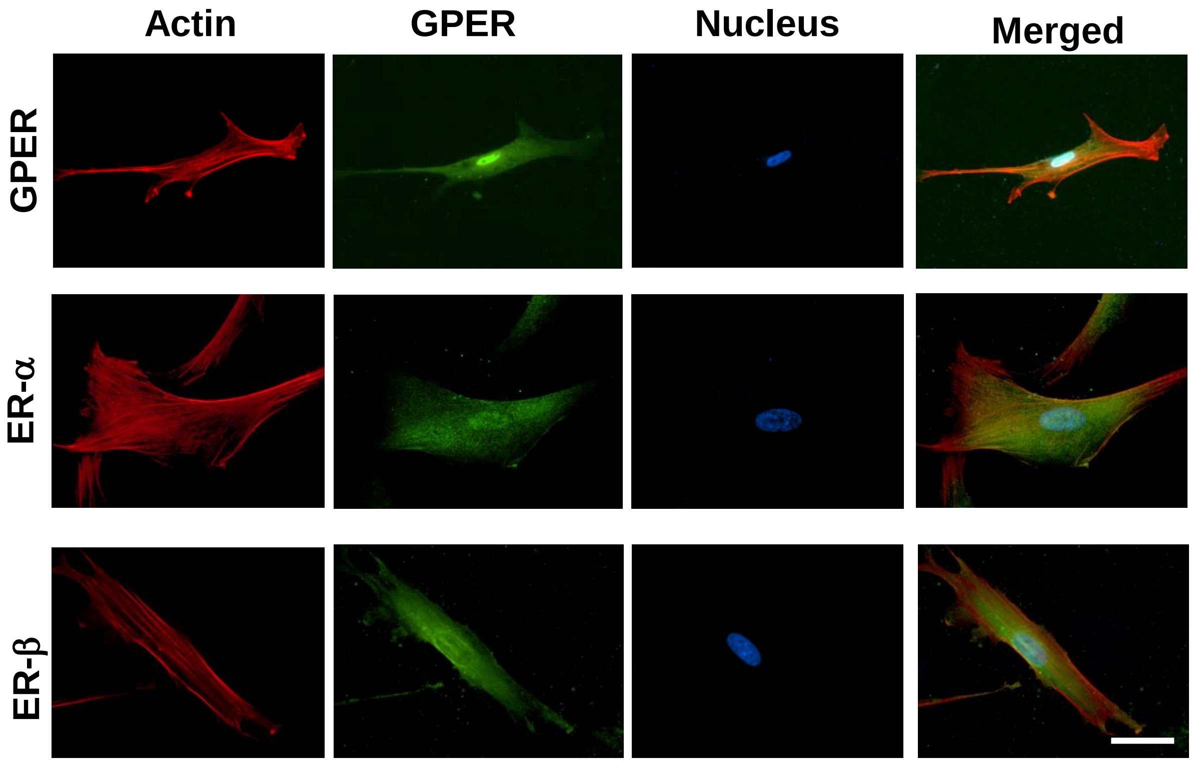

Supplementary Figure S1. Expression of G coupled protein estrogen receptor (GPER), estrogen receptor alpha (ER-α), and estrogen receptor beta (ER-β) in hepatic stellate cells HSCs. Representative images for immunofluorescence staining of HSCs, scale bar 20 µm.

Cortes *et al*.

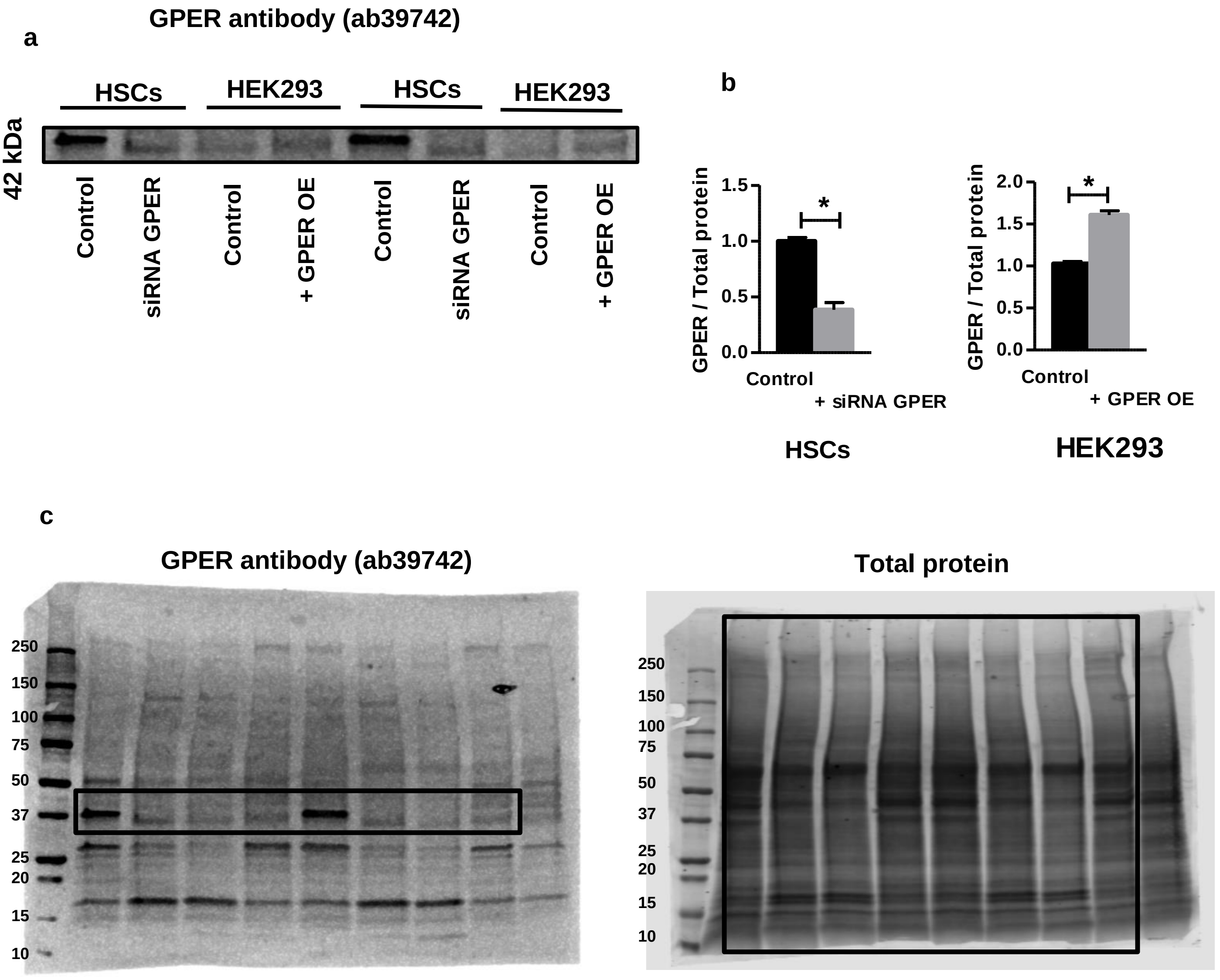

Supplementary Figure S2: **Immunoblotting experiments to validate GPER expression in HSCs and human embryonic kidney (HEK) cells (negative control). (a)** Western blot bands for GPER using abcam antibody (ab39742). OE is overexpression. **(b)** Quantification of GPER bands in HSCs and HEK293 at 42 kDa normalised to total protein and expressed relative to the control condition. **(c)** Full Membranes for Western blots of GPER and total protein.

Cortes *et al*.

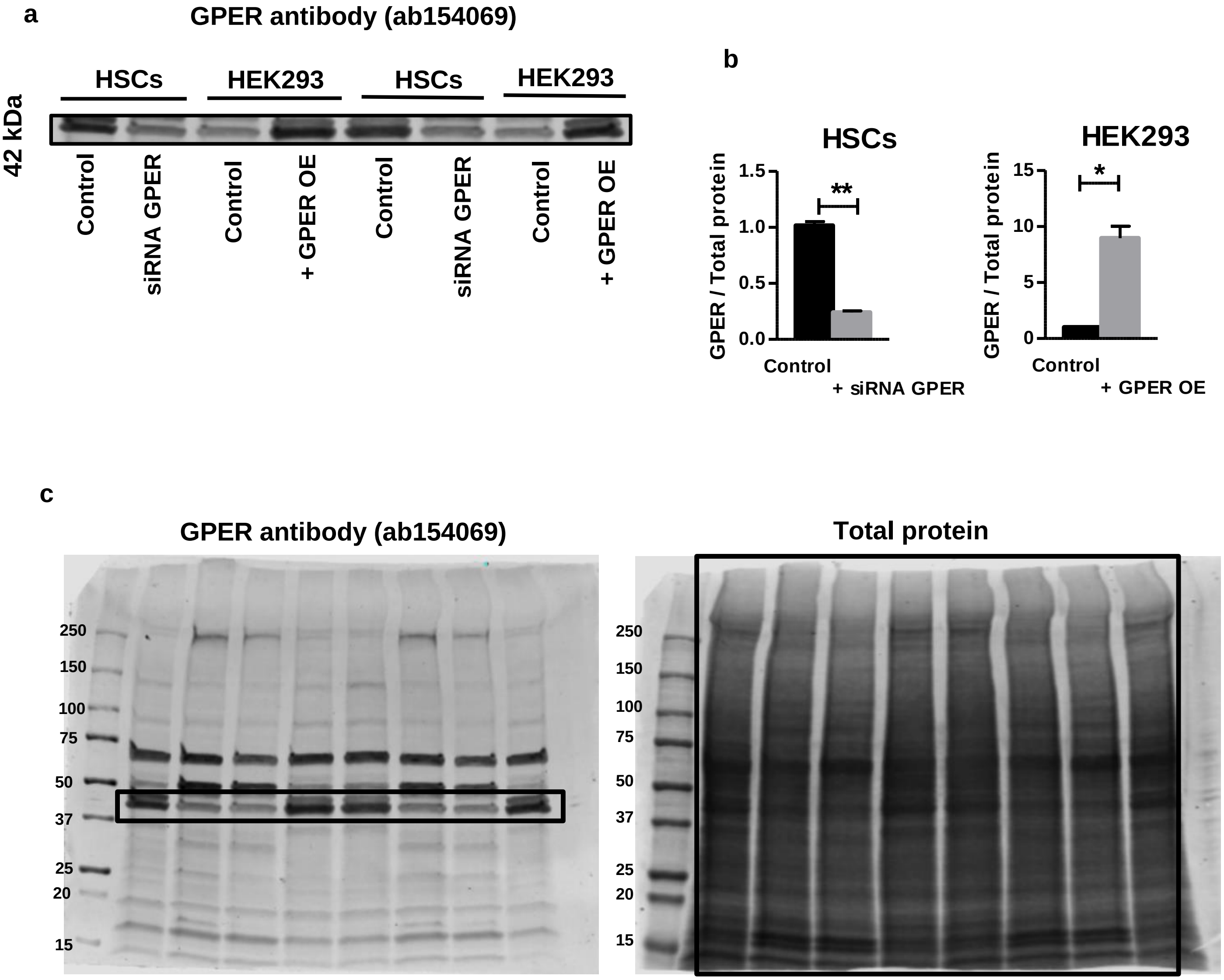

Supplementary Figure S3: **Immunoblotting experiments to validate GPER expression in HSCs and human embryonic kidney (HEK) cells (negative control). (a)** Western blot bands for GPER using abcam antibody (ab154069). OE is overexpression. **(b)** Quantification of GPER bands in HSCs and HEK293 at 42 kDa normalised to total protein and expressed relative to the control condition. **(c)** Full Membranes for Western blots of GPER and total protein.

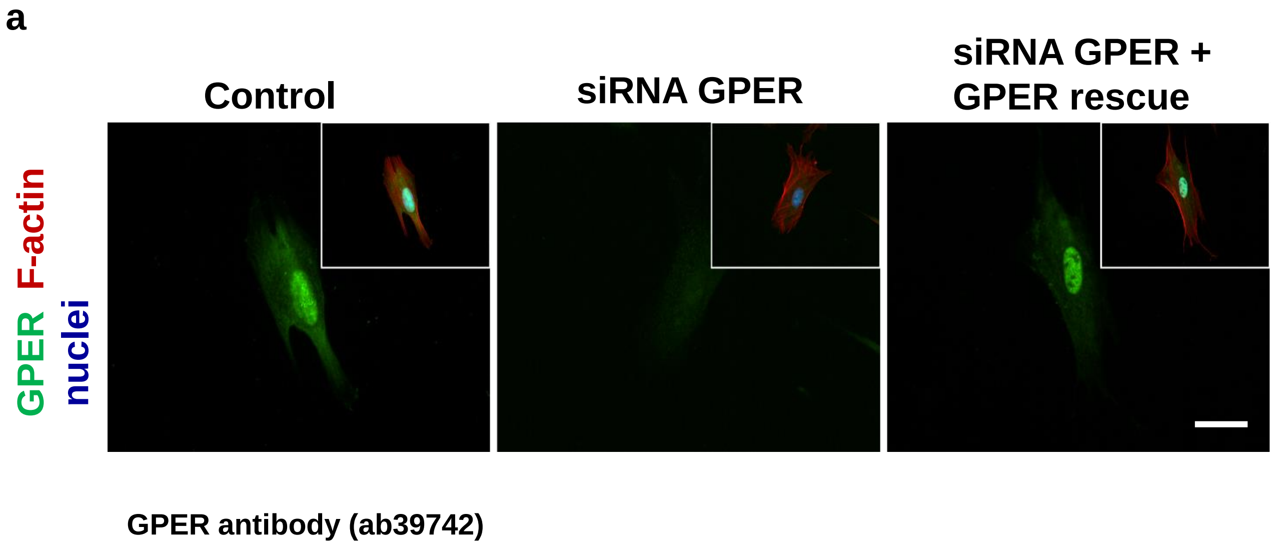

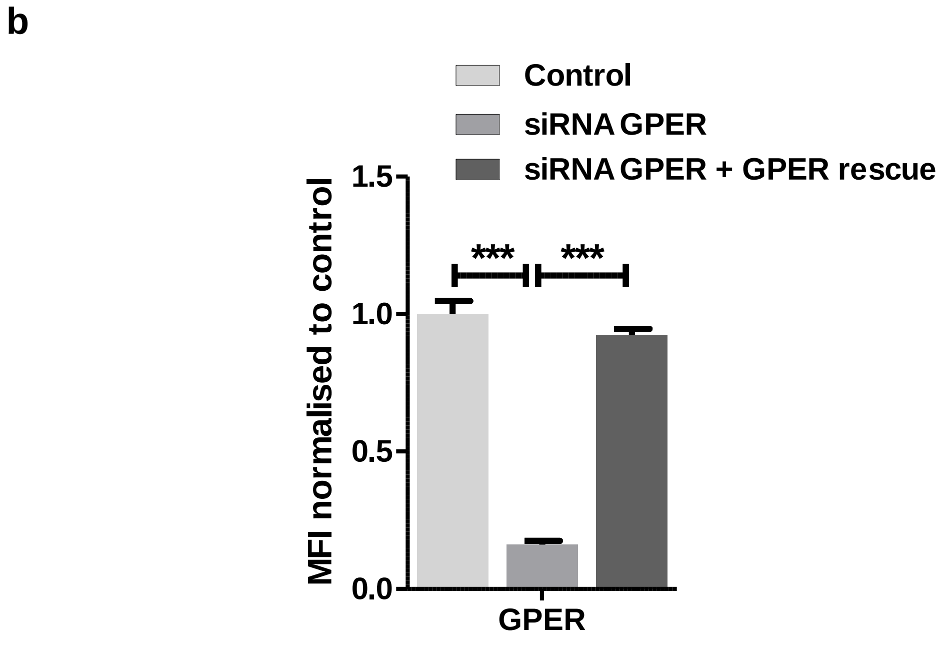

Supplementary Figure S4: Knockdown efficiency of siRNA GPER in HSCs. (a) Representative immunofluorescent images of HSCs, scale bar is 20 μm. GPER antibody used was ab35742. **(b)** Quantification of fluorescence intensity for images in panel A (n=40 control, 35 siRNA GPER, 30 siRNA GPER + GPER rescue). Three biological samples taken in three different experiments. Histogram bars represent mean ± s.e.m.;***P<0.001 (ANOVA and Tukey's post hoc test).

Cortes *et al.*

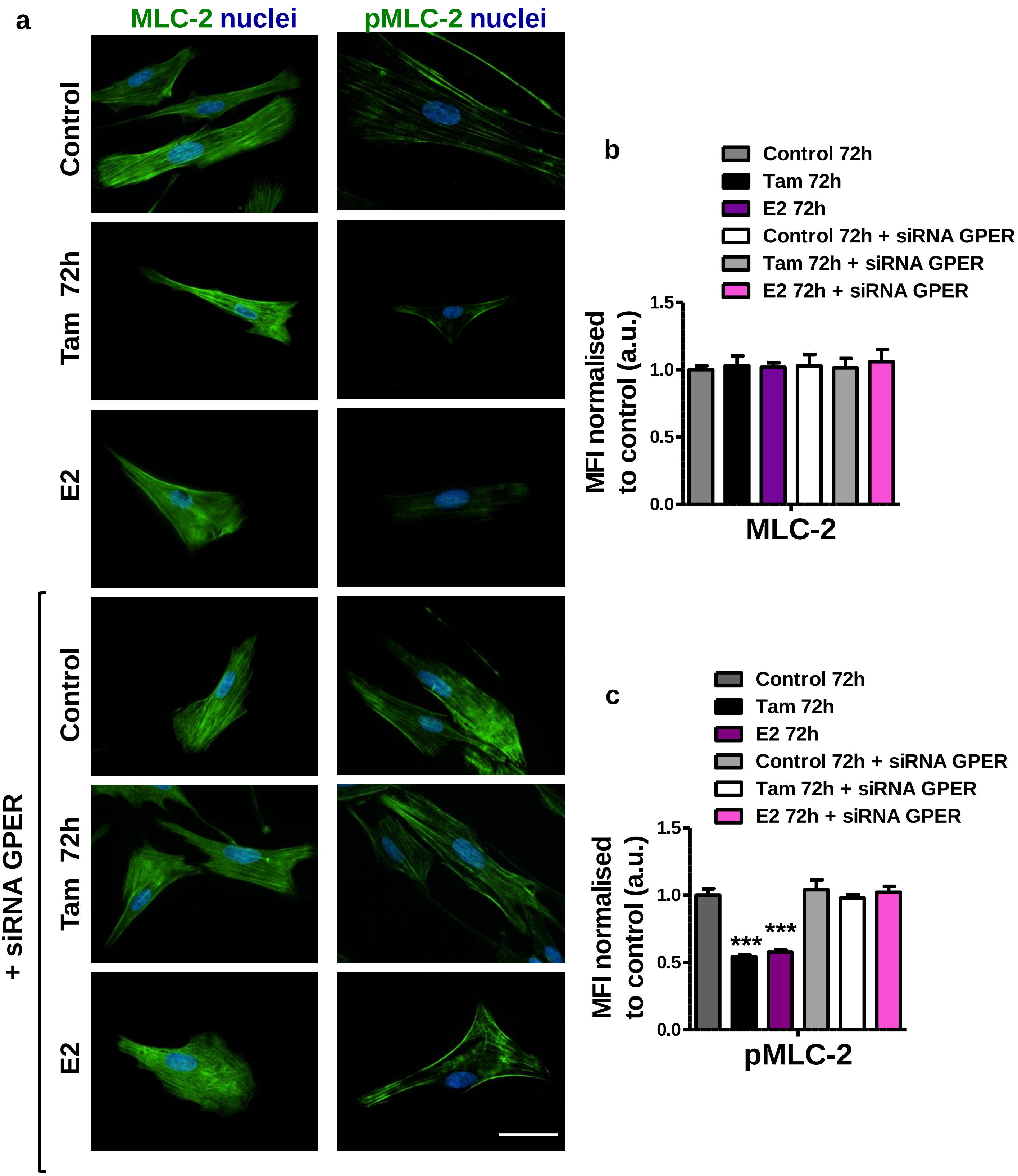

Supplementary Figure S5. Tamoxifen downregulates MLC-2 activation via GPER in hepatic stellate cells (HSCs). (a) Representative images of immunofluorescence staining. Scale bar is 20 μm. **(b, c)** Quantification of fluorescence intensity for MLC-2 and pMLC-2. MLC-2: Control, Tam (tamoxifen), E2 (β-estradiol), control + siRNA GPER, Tam + siRNA GPER, E2 + siRNA GPER n = 12, 14, 12, 11, 12, 15 cells respectively. pMLC-2: Control, Tam, E2, control + siRNA GPER, tam + siRNA GPER, E2 + siRNA GPER n = 14, 12, 12, 12, 12, 12 cells respectively. Histogram bars represent mean s.e.m., three experimental replicates. Markers denote significant differences from the control ***p < 0.001. Anova and Tukey post hoc test. Three experimental replicates.

Cortes *et al.*

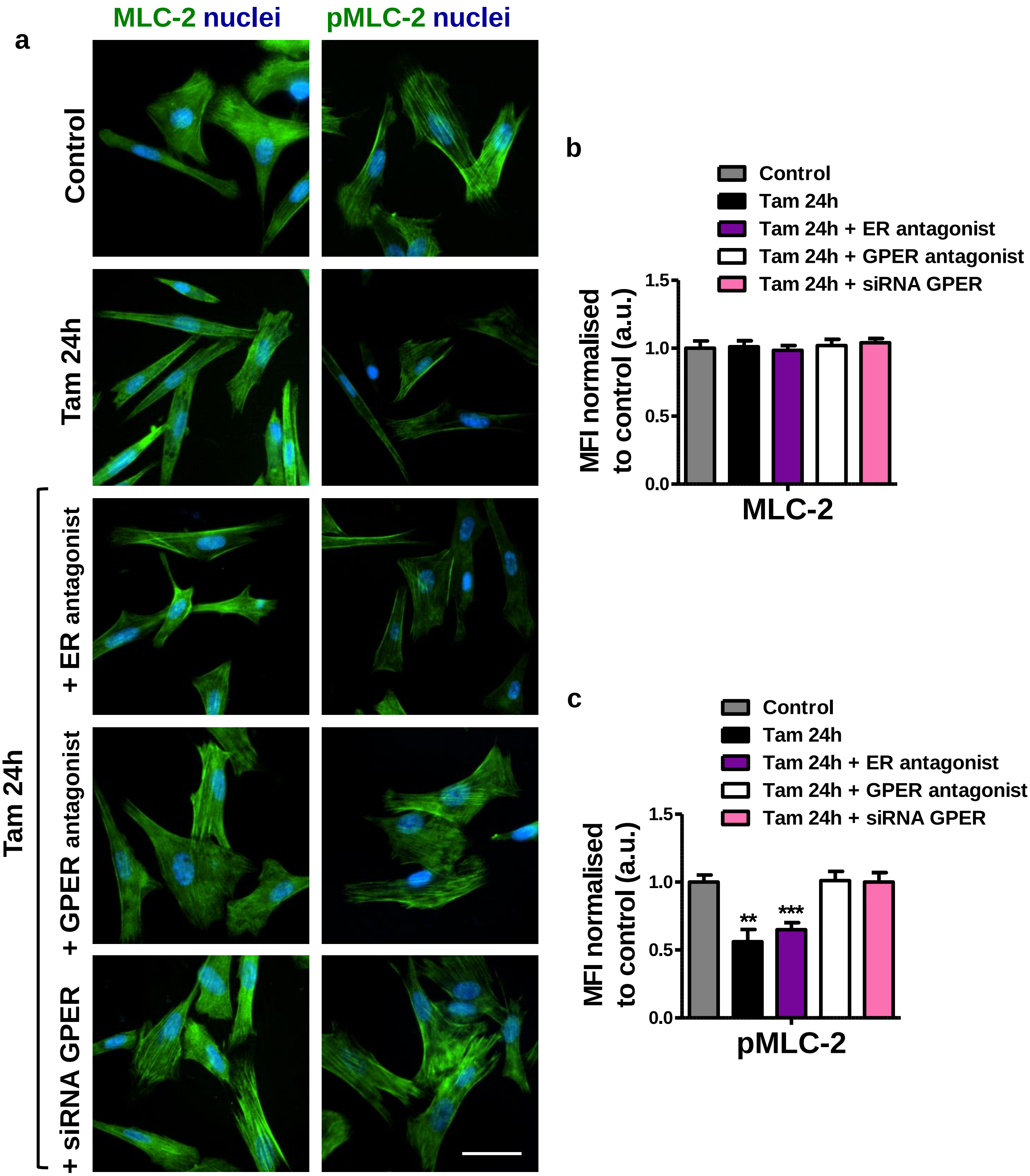

Supplementary Figure S6. 24h Tamoxifen treatment downregulates MLC-2 activation via GPER in hepatic stellate cells (HSCs). (a) Representative images of immunofluorescence staining. Scale bar is 20 µm. **(b, c)** Quantification of fluorescence intensity for MLC-2 and pMLC-2. MLC-2 number of cells: 28 Control, 20 Tam (tamoxifen), 25 Tam + ERantagonist, 27 Tam + GPERantagonist, 28 Tam + siRNA GPER. pMLC-2 number of cells: 29 Control, 24 Tam (tamoxifen), 16 Tam + ERantagonist, 22 Tam + GPERantagonist, 20 Tam + siRNA GPER. Histogram bars represent mean s.e.m., three experimental replicates. Markers denote significant differences from the control ***p < 0.001, **p < 0.005. Anova and Tukey post hoc test. Three experimental replicates.

Cortes *et al.*

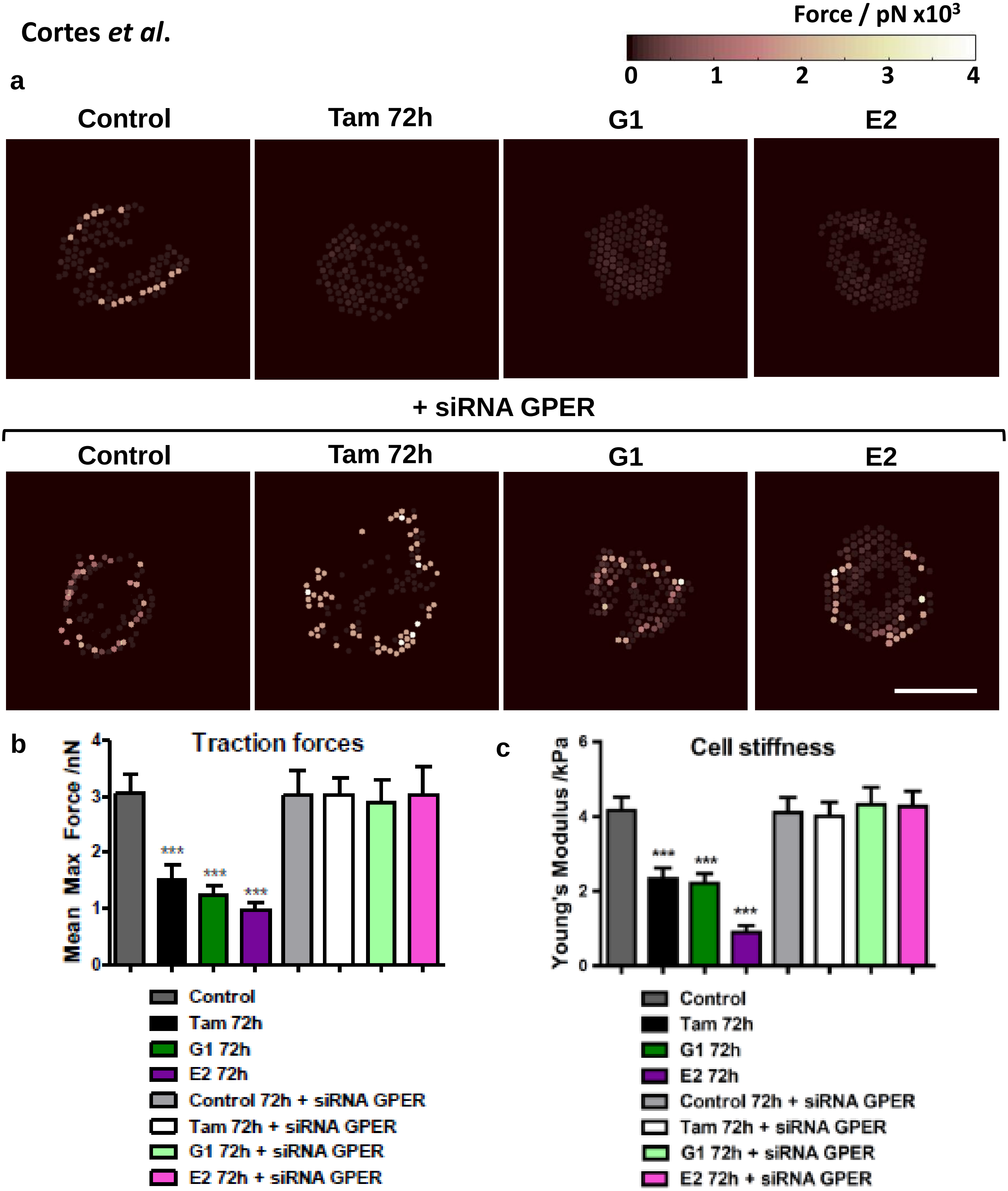

Supplementary Figure S7. Tamoxifen inhibits force generation and cell stiffness through GPER. (a) Heat maps representing force applied by HSCs on top of the pillars, scale bar = 20 µm. **(b)** Quantification of average forces applied by HSCs on pillars. For control, Tam (tamoxifen), G1 (GPER agonist), E2 (β-estradiol), control + siRNA GPER, Tam + siRNA GPER, G1 + siRNA GPER, E2 + siRNA GPER n = 53, 44, 47, 28, 37, 45, 41, 37 cells respectively. **(c)** Quantification of cell compliance with atomic force microscopy. Cantilevers used had a 15 µm polystyrene bead attached. For control, Tam, G1, E2, control + siRNA GPER, Tam + siRNA GPER, G1 + siRNA GPER, E2 + siRNA GPER n = 57, 50, 45, 36, 45, 45, 50, 43, 54 cells respectively. Mann-Whitney test for significance. Markers denote significant differences from the control ***$p < 0.001$.

a

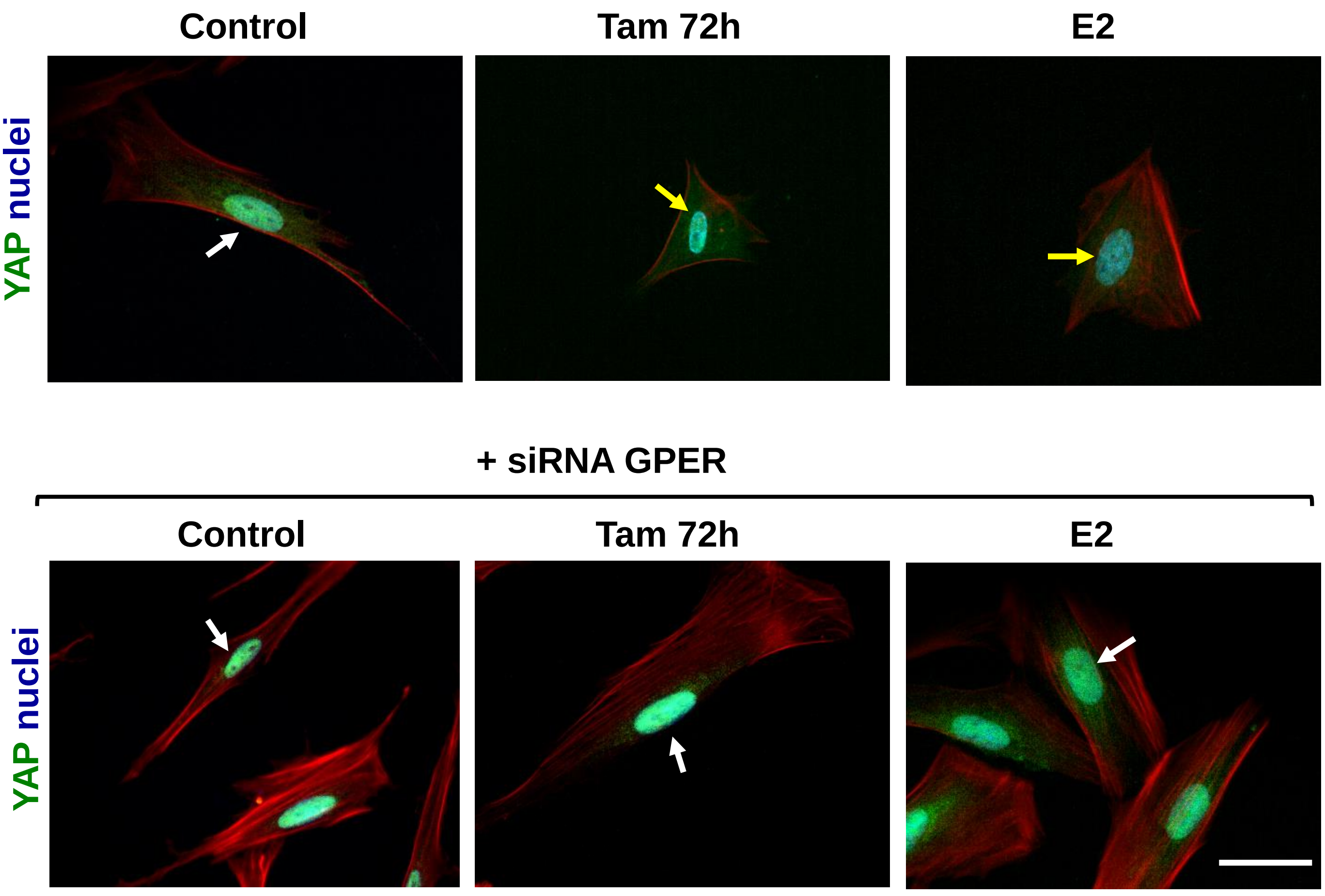

b

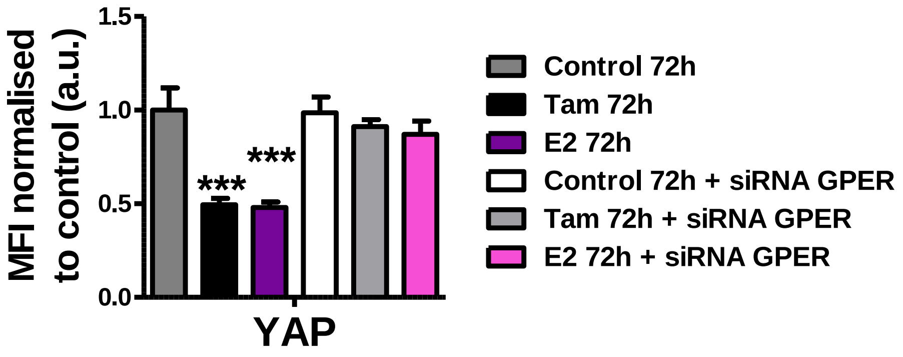

Supplementary Figure S8. Tamoxifen inhibits YAP nuclear localisation via GPER in hepatic stellate cells (HSCs). (a) Representative images of immunofluorescence staining. Scale bar is 20 μm. White arrows indicate YAP nuclear localization and yellow arrows represent nuclei devoid of YAP. **(b)** Quantification of fluorescence intensity for YAP. For control, Tam (tamoxifen), E2 (β-estradiol), control + siRNA GPER, Tam + siRNA GPER, E2 + siRNA GPER n = 12, 11, 10, 10, 11, 11 cells respectively. Histogram bars represent mean s.e.m., three experimental replicates. Markers denote significant differences from the control ***p < 0.001. Anova and Tukey post hoc test. Three experimental replicates.

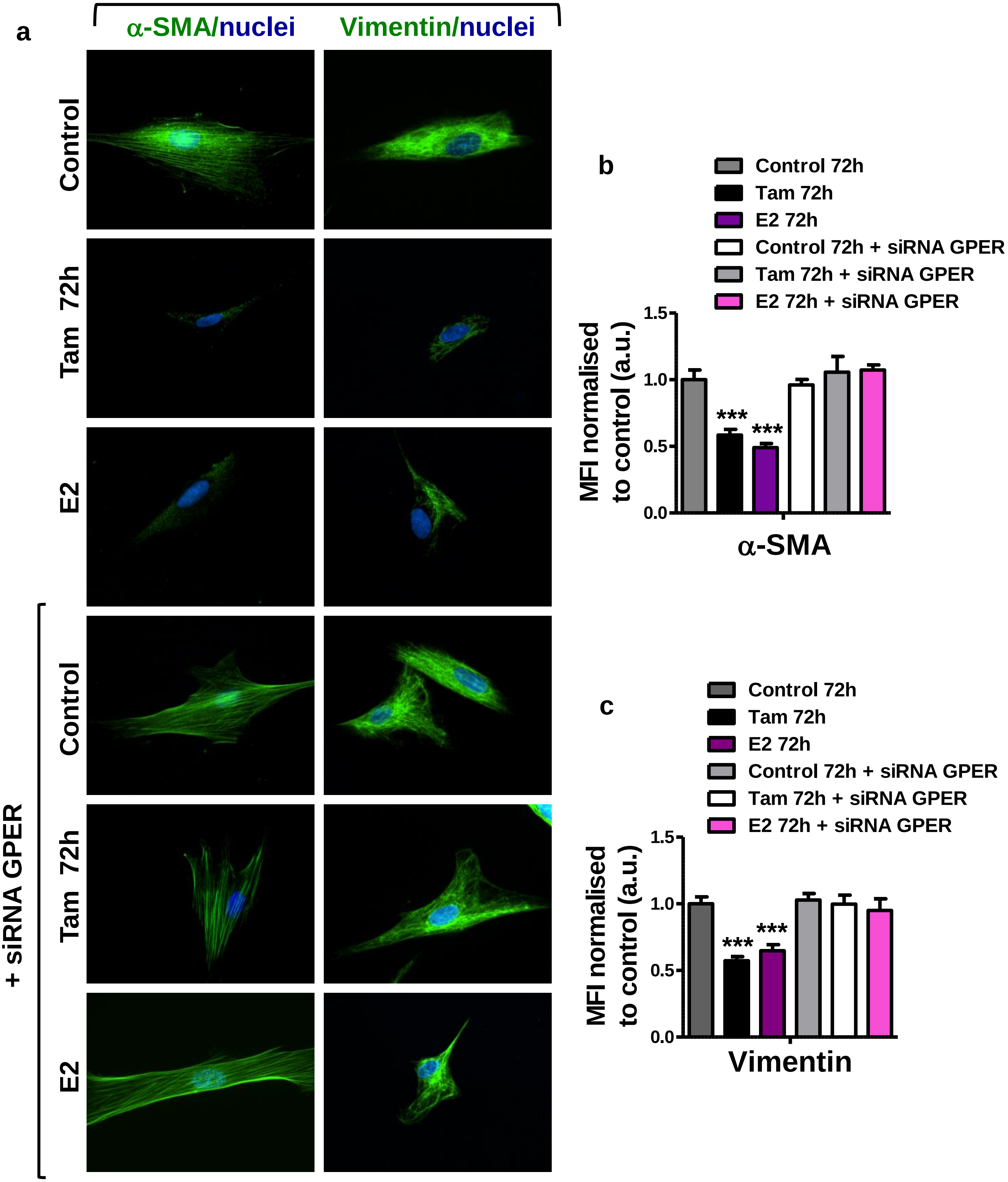

Supplementary Figure S9. Tamoxifen reduces expression of α-SMA and vimentin via GPER in hepatic stellate cells (HSCs). (a) Representative images of immunofluorescence staining. Scale bar is 20 μm. **(b, c)** Quantification of fluorescence intensity in panel a: Control, Tam (tamoxifen), E2 (β-estradiol), control + siRNA GPER, Tam + siRNA GPER, E2 + siRNA GPER n = 14, 13, 14, 13, 10, 14 cells respectively. Vimentin: Control, Tam, E2, control + siRNA GPER, Tam + siRNA GPER, E2 + siRNA GPER n = 10, 12, 12, 14, 12, 14 cells respectively. Histogram bars represent mean s.e.m., three experimental replicates. Markers denote significant differences from the control ***$p < 0.001$. Anova and Tukey post hoc test. Three experimental replicates.

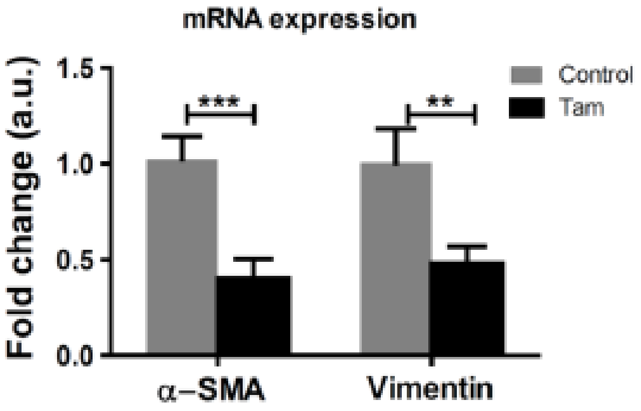

Supplementary Figure S10: 10 day Tamoxifen treatment reduces the expression of α-SMA and vimentin mRNAs in HSCs. qPCR levels of α-SMA and vimentin in HSCs, normalized to RPLP0 (60S acidic ribosomal protein P0) and relative to control. All histogram bars represent mean±sem, **P<0.01,***P<0.001. Three experimental replicates in all cases. t-test

Cortes *et al.*

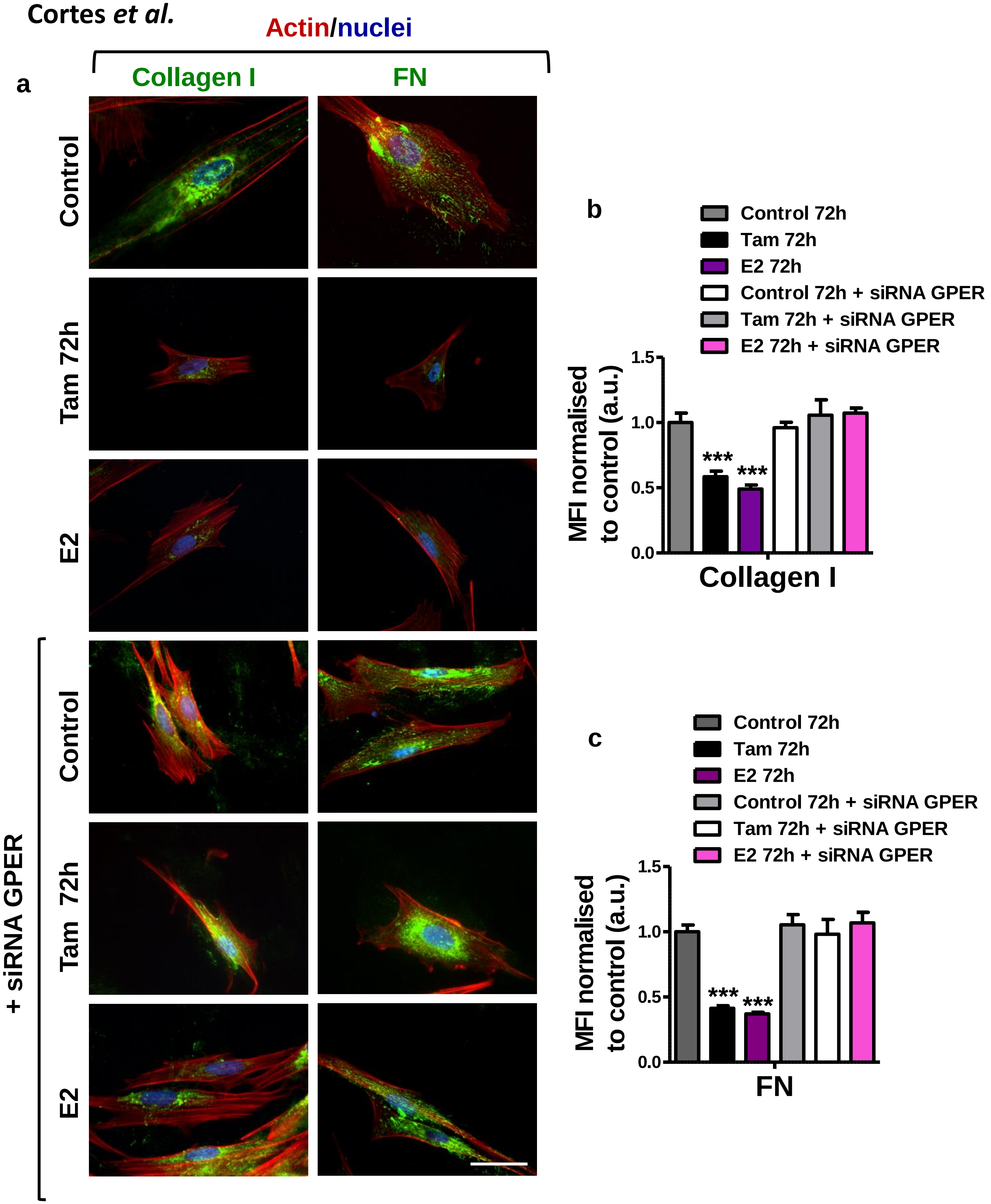

Supplementary Figure S11. Tamoxifen reduces collagen I and fibronectin (FN) expression via GPER in hepatic stellate cells (HSCs). (a) Representative images of immunofluorescence staining. Scale bar is 20 μm. **(b, c)** Quantification of fluorescence intensity for collagen I and FN. Collagen I: Control, Tam (tamoxifen), E2 (β-estradiol), control + siRNA GPER, Tam + siRNA GPER, E2 + siRNA GPER n = 14, 13, 14, 13, 10, 12 cells respectively. FN: Control, Tam, E2, control + siRNA GPER, Tam + siRNA GPER, E2 + siRNA GPER n = 10, 12, 13, 12, 14, 15 cells respectively. Histogram bars represent mean s.e.m., three experimental replicates. Markers denote significant differences from the control ***p < 0.001. Anova and Tukey post hoc test. Three experimental replicates.

Cortes *et al*.

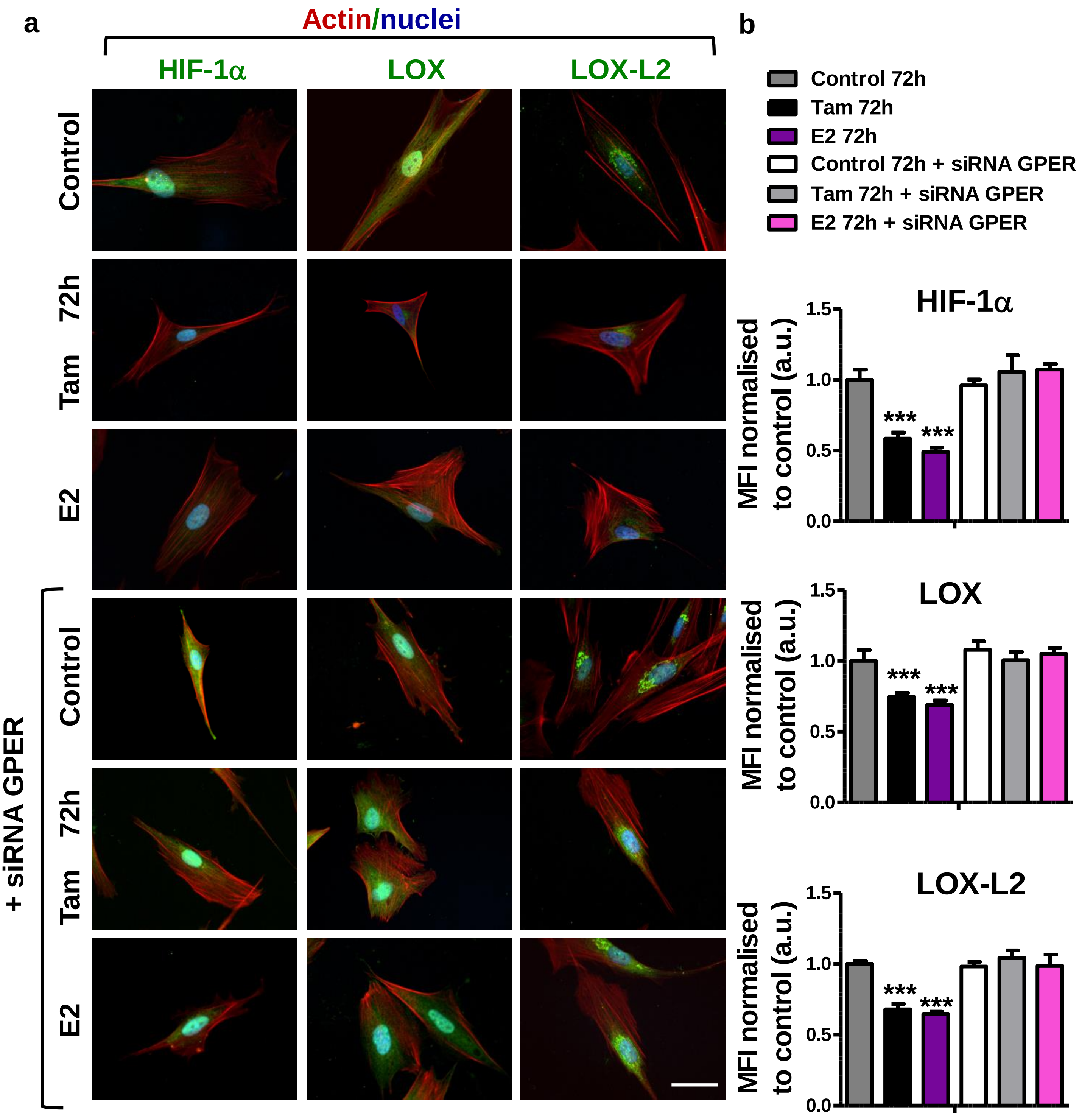

Supplementary Figure S12. Tamoxifen reduces expression of HIF-1α, LOX, and LOX-L2 via GPER in hepatic stellate cells (HSCs). (a) Representative images of immunofluorescence staining. Scale bar is 20 μm. **(b)** Quantification of fluorescence intensity. HIF-1α: Control, Tam (tamoxifen), E2 (β-estradiol), control + siRNA GPER, Tam + siRNA GPER, E2 + siRNA GPER n = 14, 13, 14, 13, 10, 12 cells respectively. LOX: Control, Tam, E2, control + siRNA GPER, Tam + siRNA GPER, E2 + siRNA GPER n = 12, 15, 17, 12, 13, 14 cells respectively. LOX-L2: Control, Tam, E2, control + siRNA GPER, Tam + siRNA GPER, E2 + siRNA GPER n = 11, 12, 14, 10, 11, 12 cells respectively Histogram bars represent mean s.e.m., three experimental replicates. Markers denote significant differences from the control ***$p < 0.001$. Anova and Tukey post hoc test. Three experimental replicates.

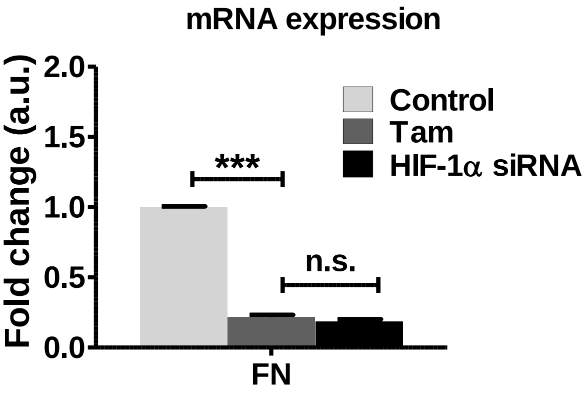

Supplementary Figure S13: Tamoxifen effect on fibronectin (FN) is mediated by HIF-1α. qPCR levels of FN in HSCs, normalized to RPLP0 (60S acidic ribosomal protein P0) and relative to control. Histogram bars represent mean±sem,***$P<0.001$. Three experimental replicates, Anova and Tukey's test.

a

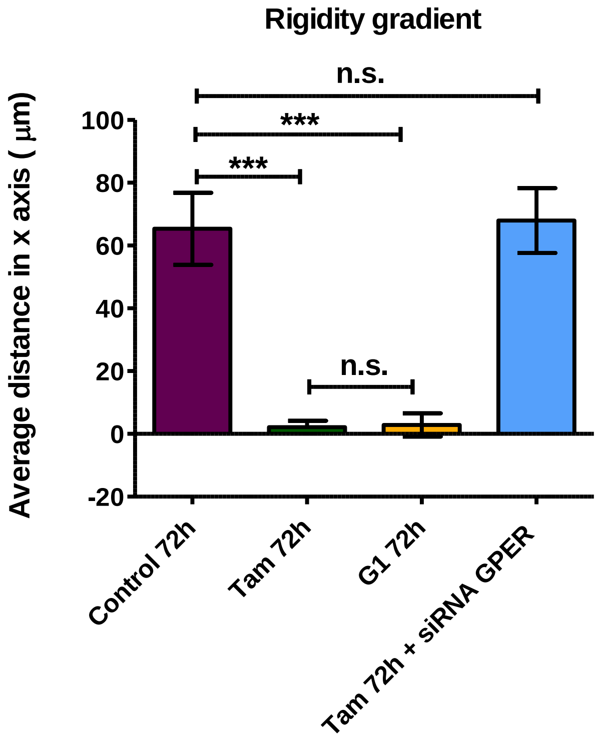

b

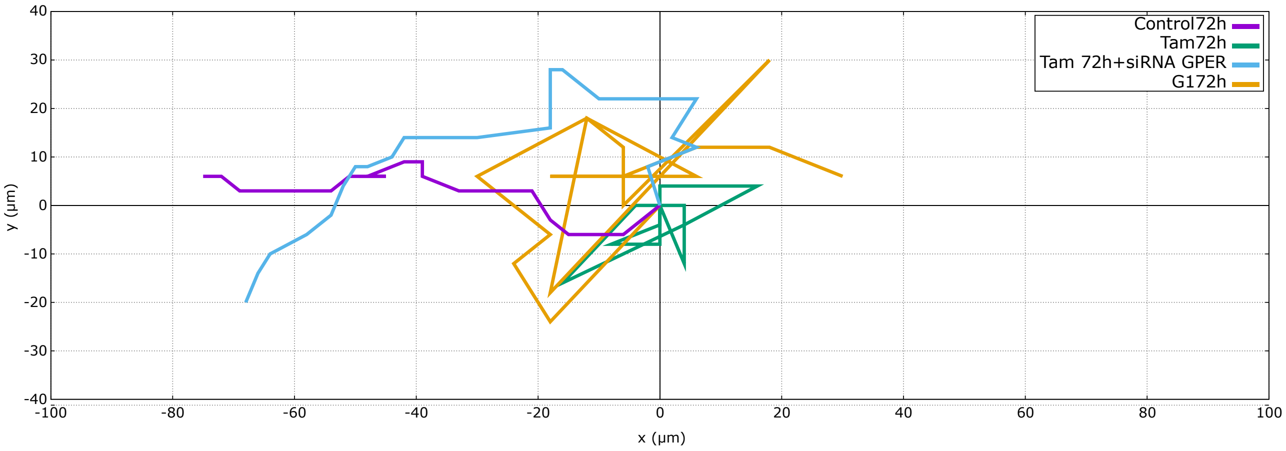

Supplementary Figure S14. Tamoxifen treatment inhibits HSCs durotaxis via GPER signalling. **(a)** Average cell movement distance on the soft-stiff rigidity gradient compared to single rigidity soft and stiff substrates presented as an average displacement (positive values indicate directed movement towards stiff substrate, negative values towards soft substrate and 0 indicates random movement. n = 3 independent experiments. **(b)** Representation of the average displacements of HSCs. Quantification was done for 66 cells. Three experimental replicates in all cases. Results are expressed as mean ± sem. Anova and Tukey's post hoc tests were used for the analysis.
