# Supplementary material for: Tamoxifen mechanically deactivates hepatic stellate cells via the G protein-coupled estrogen receptor
Source: Oncogene. 2018 Dec 21;38(16):2910–22. doi: 10.1038/s41388-018-0631-3 (PMC6755965; doi:10.1038/s41388-018-0631-3)
Supplement: Supplementary file 3 — Supplementary methods information [file 41388_2018_631_MOESM3_ESM.docx]

**Supplementary methods information**

**Analysis of gene expression using TCGA data**

Liver Hepatocellular Carcinoma (LIHC) data (492 samples) from the Cancer Genome Atlas (TCGA) was used to validate GPER expression levels in distinct subtypes of HCC.  The Cancer Genomic Data Server (CGDS) was accessed on 18 January 2018 through the CGDS-R^a^ package. The R^b^ statistic program was used to retrieve patients with GPER expression from RNA-Seq data using the CGDS-R package for querying the Cancer Genomic Data Server (CGDS). GPER expression levels were extracted for 373 patients and compared with the expression of following genes: Vimentin, aSMA, CTGF, ANKYRIN, YAP, HIF1a, LOX, LOX-L2, Fibronectin, Collagen-I. Pairwise comparisons were performed using the t-tests with pooled SD with R statistic program. (a) Anders Jacobsen (2017). cgdsr: R-Based API for Accessing the MSKCC Cancer Genomics Data Server (CGDS). R package version 1.2.6. <https://CRAN.R-project.org/package=cgdsr>. (b) R Core Team (2017). R: A language and environment for statistical computing. R Foundation for StatisticalComputing, Vienna, Austria. URL <https://www.R-project.org/>

**Traction forces using elastic pillars**

Pillar arrays were created by mixing polydimethylsiloxane (PDMS) and a curing agent (Sylgard 184) in a 10:1 ratio (giving a Young’s modulus of 2 MPa)(1) and pouring this mixture into a pillar mould with holes of 5 µm depth. This was incubated at 70 degrees for 12 hours for the PDMS to set. Once separated from the mould, PDMS pillars were coated with fibronectin (10 µl/mL PBS) for 1.5 hr at 37 degrees. The solution surrounding the pillars was replaced with PBS for washing, and then replaced by cell medium. Cells were trypinised and resuspended in culture medium and seeded onto the pillars, then incubated for 1 hr at 37 degrees 5% CO_2_ before transfer to a Nikon Ti-e microscope in a 37 degree temperature controlled chamber. Videos of different were taken at 1 frame per second for 60 seconds, and each pillar mould was imaged for a maximum time of 30 mins to ensure cell viability. The position of each pillar in the time-lapse videos was tracked using a custom MATLAB program to track the centre of a point spread function of the intensity of the pillars across all frames. By selecting a location free of cells, tracking of a small set of pillars allowed a measurement of the stage drift to be obtained and corrected for in the data set. The time-dependent displacement of a given pillar was obtained by subtracting the initial position of the pillar (zero force) from the position in a given frame. Traction forces were obtained by multiplying the pillar displacements by the pillar stiffness. Pillar stiffness is related to pillar height by the equation $F=k\Delta x=(\frac{3}{64}\pi E\frac{D^{4}}{L^{3}})\Delta x$, where F = force, k = pillar spring constant, Δx = pillar displacement, E = PDMS Young’s modulus, D = pillar diameter and L = pillar length(2). The maxima for each pillar were found to obtain the average peak force across the cell.

**Cell mechanosensing**

HSCs (control or tamoxifen treated) were incubated with 4.5 μm FN-coated magnetic beads coated for 30 minutes and then subjected to a pulsatile force regimen applied with magnetic tweezers, consisting of a 3 s, 1 nN pulse of force, followed by a 4 s period of rest, repeated for 12 total pulses over a 100 s time course. The ability of the cells to sense and respond to the applied tension was examined from the rapid cell stiffening response evident by the progressive decrease in amplitude of the bead movement.

**Quantification and analysis of durotaxis on polyacrylamide hydrogels**

Durotaxis of cells was analysed with a Nikon Ti-Eclipse microscope using a 20x objective. After cell seeding onto dual-rigidity hydrogels, samples were transferred to microscope culture chamber (37°C, 5% CO2) and gently submerged in 5 mL of growth media. The rigidity boundary was identified through yellow-green fluorescence of FluoSpheres. Regions of interest (ROI) across the sample were stitched together using NIS elements software to generate a representative image of the hydrogel surface. x- and y-axis were used to define these ROI within the ‘soft’, ‘stiff’ and ‘rigidity gradient’ regions of the hydrogel, whilst the z-axis was used to focus the camera onto the surface plane of the gel. A period of 1-2 hours was set to allow cells to fully attach to gel surface before time-lapse phase contrast images were taken every 10 minutes for 5.5 hours within each designated ROI. Coordinates and distances of cell movement were calculated using the Fiji “Manual Tracking” plugin.

**Atomic force microscopy**

Measurements of cell compliance were conducted on a Nanowizard-1 (JPK Instruments, Berlin, Germany) atomic force microscope operating in force spectroscopy mode mounted on an inverted optical microscope (IX-81; Olympus, Tokyo, Japan). Atomic force microscopy (AFM) pyramidal cantilevers (MLCT; Bruker, Camarillo, CA, USA) with a spring constant of 0.03 N/m were used with a 15 µm polystyrene bead attached. Before conducting measurements, cantilever sensitivity was calculated by measuring the force–distance slope in the AFM software on an empty petri dish region. For each cell analysed, force curves were acquired at an approach speed of 5 µm/s and a maximum set point of 1 V. The force–distance curves were used to calculate elastic moduli in the AFM software through the application of the Hertz contact model(3).

**G-LISA assay for RhoA**

The intracellular amounts of total RhoA and RhoA-GTP were determined by using the total RhoA ELISA and G proteins-linked (G-LISA) assays (Cytoskeleton, Inc., Denver, CO, USA) according to the manufacturer’s instructions. Briefly, cells were washed with cold PBS and homogenized gently in ice-cold lysis buffer. 20 μl was removed for protein quantification in order to adjust sample concentration to 0.5 mg/ml. After adding an equal volume of binding buffer, triplicate assays were performed using 1.5 μg protein per well. Samples were incubated for 30 minutes and then washed three times with washing buffer. Antigen-presenting buffer was added for two minutes before removal; samples were then incubated with 1:250 dilution of anti-RhoA antibody at room temperature for 45 minutes, washed three times, and incubated with secondary antibodies for another 45 minutes. HRP detection reagent was added and signal was read by measuring absorbance at 490 nm using a microplate spectrometer.

**Statistical analysis**

All statistical analyses were conducted with the Prism graphical software (GraphPad, Software). Data were generated from multiple repeats of different biological experiments to obtain the mean values and s.e.m displayed throughout. P values have been obtained through t-tests on paired or unpaired samples with parametric tests used for data with a normal distribution and non-parametric tests conducted via the Mann–Whitney test where data had a skewed distribution. Significance for the t-tests was set at P<0.05 where graphs show significance through symbols (*P<0.05; **P<0.01; ***P<0.001).

**References for the supplementary methods section:**

1. Chronopoulos A, Robinson B, Sarper M, Cortes E, Auernheimer V, Lachowski D, et al. ATRA mechanically reprograms pancreatic stellate cells to suppress matrix remodelling and inhibit cancer cell invasion. Nature communications. 2016;7:12630.

2. Schoen I, Hu W, Klotzsch E, Vogel V. Probing cellular traction forces by micropillar arrays: contribution of substrate warping to pillar deflection. Nano letters. 2010;10(5):1823-30.

3. Harris AR, Charras GT. Experimental validation of atomic force microscopy-based cell elasticity measurements. Nanotechnology. 2011;22(34):345102.
